# Circ-Tulp4 promotes β-cell adaptation to lipotoxicity by regulating soat1 expression

**DOI:** 10.1530/JME-20-0079

**Published:** 2020-09-11

**Authors:** Liting Wu, Li Xiong, Jin Li, Zishan Peng, Luyao Zhang, Peijie Shi, Yingying Gong, Haipeng Xiao

**Affiliations:** 1 Department of Endocrinology and Metabolism, The First Affiliated Hospital of Sun Yat-sen University, Guangzhou, Guangdong, China; 2 Department of Geriatrics, The First Affiliated Hospital of Sun Yat-sen University, Guangzhou, Guangdong, China

**Keywords:** type 2 diabetes, circular RNA, β-cell proliferation, β-cell lipotoxicity

## Abstract

This study aimed to identify circular RNAs differentially expressed in the islets of type 2 diabetes (T2DM) models and clarify their roles in the control of β-cell functions. Circular RNAs dysregulated in the islets of diabetic db/db mice were identified by high-throughput RNA sequencing. Then, the expression level of the selected circular RNA circ-Tulp4 was confirmed by real-time PCR in the islets of diabetic models and Min6 cells. MTS, EdU, western blot, flow cytometric analysis, and luciferase assay were performed to investigate the impact of circ-Tulp4 on β-cell functions. This study identified thousands of circular RNAs in mouse pancreatic islets. The circ-Tulp4 level significantly decreased in the diabetic models and altered in the Min6 cells under lipotoxic condition. The modulation of circ-Tulp4 level in Min6 cells regulated cell proliferation. Furthermore, an interaction was demonstrated between circ-Tulp4 and miR-7222-3p, which suppressed the expression of cholesterol esterification-related gene, sterol O-acyltransferase 1 (*SOAT1*). The accumulation of soat1 activated cyclin D1 expression, thus promoting cell cycle progression. These findings showed that circ-Tulp4 regulated β-cell proliferation via miR-7222-3p/soat1/cyclin D1 signaling. Our research suggested that circ-Tulp4 might be a potential therapeutic intervention for T2DM. Besides, soat1 might be important for β-cell adaptation to lipotoxicity.

## Introduction

Pancreatic β cells are specialized insulin-secreting cells responsible for the control of blood glucose levels (Ferrannini & Mari 2004). β-Cell mass reduction and/or dysfunction can lead to hyperglycemia and the development of diabetes (Heit * et al.* 2006). Type 2 diabetes (T2DM) is characterized by insufficient β-cell function under obesity-induced insulin resistance (Kahn *et al.* 2006). In the prediabetic condition, β cells accelerate their proliferation rates to compensate for the β-cell loss caused by apoptosis (Prentki & Nolan 2006, Montanya & Téllez 2009). However, prolonged exposure to high concentrations of glucose and free fatty acids increase β-cell apoptosis and inhibit proliferation, eventually reducing β-cell mass and inducing T2DM manifestation (Busch *et al.* 2005, Prentki & Nolan 2006, Schofield & Sutherland 2012). Therefore, a better understanding of the molecular mechanisms underlying β-cell adaptation is vital for designing new therapeutics for T2DM.

Previous transcriptome analyses have unveiled that a large number of non-coding RNAs are expressed in β cells (Nesca * et al.* 2013, Motterle * et al.* 2017, Stoll *et al.* 2018, Xiong * et al.* 2020). Available evidence indicates that small RNAs (miRNA) and long non-coding RNAs play key roles in regulating β-cell functions (Guay *et al.* 2012, Nesca *et al.* 2013, Motterle *et al.* 2017). However, experimental data linking circular RNAs (circRNAs) to β-cell functions are limited.

Recently, the advancement of biotechnology has increased the understanding of the expression and function of circRNAs (Suzuki *et al.* 2006, You *et al.* 2015, Xu *et al.* 2017). circRNAs contain mainly exonic sequences, which are back-spliced by spliceosome mediation (Barrett *et al.* 2015, Starke *et al.* 2015). Because of the circular secondary structure, circRNAs are more stable than linear transcripts (Suzuki *et al.* 2006). Furthermore, the expression of circRNAs generally exhibits cell-type and tissue-specific patterns (Salzman *et al.* 2013, Enuka *et al.* 2016). circRNAs have been frequently reported to participate in diverse gene-regulatory mechanisms, and their dysregulations have been implicated in health and disease (Zhang *et al.* 2013, 2018*b*, Du *et al.* 2016, 2017). In diabetes, circHIPK3 and CDR1as have been shown to regulate β-cell proliferation and insulin secretion in mouse islets by sponging multiple miRNA and modulating the activity of miRNA on other target genes (Xu *et al.* 2015, Stoll *et al.* 2018). However, the functional characterization of most discovered circRNAs in β cells remains to be elucidated.

In this study, whole transcriptome sequencing was used to explore the global variations in islet transcriptomes in db/db diabetic mice and db/m healthy control mice. Hundreds of circRNAs displayed expression changes, and circ-Tulp4 showed significant downregulation in diabetic mouse islets and Min6 cell lines under lipotoxic condition. Further experiments indicated that modulating the expression of circ-Tulp4 in Min6 cells desensitized β cells to lipotoxicity. This study proved that circ-Tulp4 promoted β-cell function by sponging miR-7222-3p and regulating the expression of cholesterol esterification-related gene, sterol O-acyltransferase 1 (*SOAT1*) and cyclin D1 signaling.

## Materials and methods

### Animal experiments

Animal experiments were conducted following relevant Chinese institutional laws and guidelines and were approved by the local ethics committee of Sun Yat-sen University. All mice were obtained from the Model Animal Research Center of Nanjing University (Nanjing, China).

Five-week-old male db/db diabetic mice (BKS.Cg-Dock7m+/+Leprdb/Nju) and age-matched non-diabetic male littermate db/m mice were placed on a normal diet. After 5 weeks, they were sacrificed for islets isolation. Five-week-old male C57BL/6J mice were placed on a normal control diet (NFD) (D12450J, Research Diets, New Brunswick, NJ, USA) or high-fat diet (HFD) (D12492, Research Diets) for 8 months until they were sacrificed for islets isolation.

The mice were killed with phenobarbital sodium (40 mg/100 g body weight) and 3 mL of PBS containing 0.4 mg/mL collagenase P (Roche) was immediately injected into the bile duct. Islets were isolated by collagenase digestion followed by purification on Histopaque (Sigma Aldrich) density gradient and used directly for RNA extraction.

### Culture and treatment of cells

Min6 cells, obtained from the American Type Culture Collection (ATCC), were cultured in a mixture containing DMEM, 15 (v/v) fetal bovine serum (FBS), 100 mg/mL streptomycin, 100 U/mL penicillin, and 5 μL/L β-mercaptoethanol. HEK293 cells were obtained from ATCC and cultured in DMEM supplemented with 10 (v/v) FBS. Passage numbers of Min6 cells used for experiments were 5–20, while HEK 293 cells were 3–5.

For palmitate acid (PA) treatment, 0.5 mmol PA (Sigma) was dissolved in 5 (w/v) BSA (5 mL) and 0.1M NaOH (5 mL) to make a stock solution (50 mM). The lipotoxic condition was induced with PA (0.5 mM) for 24 h. The control cells were exposed to 0.1 (w/v) BSA at the same time.

For cytokine treatment, Min6 cells were exposed to cytokine cocktails consisting of IL-1β, TNF-α, and IFN-γ for 24 h (Sino Biological, China); cytokine cocktail 1 consists of IL-1β (5 ng/mL), TNF-α (25 ng/mL) and IFN-γ (25 ng/mL); cytokine cocktail 2 consists of IL-1β (1 ng/mL), TNF-α (20 ng/mL) and IFN-γ (20 ng/mL). The culture medium treated cells were used as control.

### Whole-transcriptome sequencing

The Hiseq Sequencer platform was used for whole-transcriptome sequencing. The library for sequencing was constructed by removing rRNA from the total RNA of six samples (3 db/m and 3 db/db mice). Clean reads were obtained by filtering the adaptor sequences, N bases, and low-quality reads. Then, the reads were mapped to the *Musmusculus* genome (Build version mm10) using the TopHat program (V2.0.14). The DESeq algorithm was employed to analyze differential expressed genes (Anders & Huber 2010). The ACFS circRNA prediction pipeline was used for circRNA identification (You & Conrad 2016). The Gene Ontology and pathway analysis were performed to elucidate biological implications and significant pathways of differentially expressed genes (Ashburner *et al.* 2000, Draghici *et al.* 2007). The miRanda tool and RNAhybrid algorithm were used for discovering competing endogenous RNA (ceRNA) relation (Grimson *et al.* 2007, Friedman *et al.* 2009, Garcia *et al.* 2011). Sequence data is available at http://www.ncbi.nlm.nih.gov/geo/query/acc.cgi?acc=GSE138096.

### Modulation of gene levels in Min6 cells

Transfection of microRNA mimics or inhibitors (RiboBio) or siRNAs (RiboBio) to Min6 cells was achieved using the Lipofectamine 3000 reagent (Invitrogen) according to the manufacturer’s protocols. The final concentration was 50 nM. Two siRNAs were used simultaneously to avoid off-target effects. The genes were overexpressed via infection with purified Adeno-associated virus (AAV) in the culture medium. The sequence fragment of circRNA or mRNA was amplified, sequenced, and inserted into AAV pK4ssAAV-GFP plasmids to construct the overexpression vectors (Geneseed). The cells were cultured for 48 h post-gene modulation and processed for subsequent experiments.

### Western blot analysis

Western blot analysis was performed according to a method described in a previous study (Nesca *et al.* 2013). The membranes were incubated with appropriate diluted primary antibodies overnight and secondary antibodies. The bands were visualized using a chemiluminescent substrate (Invitrogen), and the intensities were quantified using the ImageJ software.

Primary antibodies targeting ki67 (cat# 2586 Cell Signaling Technology), cleaved caspase-3 (cat# 9661S Cell Signaling Technology), soat1 (cat# PA5-76906 Invitrogen), cyclin D1 (cat# ab.134175 Abcam), β-actin (cat# 4967 Cell Signaling Technology), β-tubulin (cat# 2128 Cell Signaling Technology) were used in this study.

### Measurement of insulin secretion

Glucose-stimulated insulin secretion (GSIS) assay was performed as previously described (Xiong *et al.* 2020). To normalize the amount of secreted insulin, the cell number was recalculated, and the insulin secretion was expressed as the concentration of insulin (ng/mL) secreted per 5 × 10^4^ cells.

### Data statistical analysis

All experiments were repeated at least three times. The results were expressed as either individual data points or mean ± s.e.m. unless otherwise indicated. The data were compared using the Student’s *t-*test between two groups or ANOVA for more than two groups, followed by Dunnett’s multiple comparisons. A *P* value < 0.05 was considered statistically significant, which was indicated in the figures.

Methods are shown in the Supplementary Materials and methods (see section on supplementary materials given at the end of this article) in detail.

## Results

### RNA-seq analysis of islet circRNAs and mRNA profiling in db/db mice

**Figure 1:**
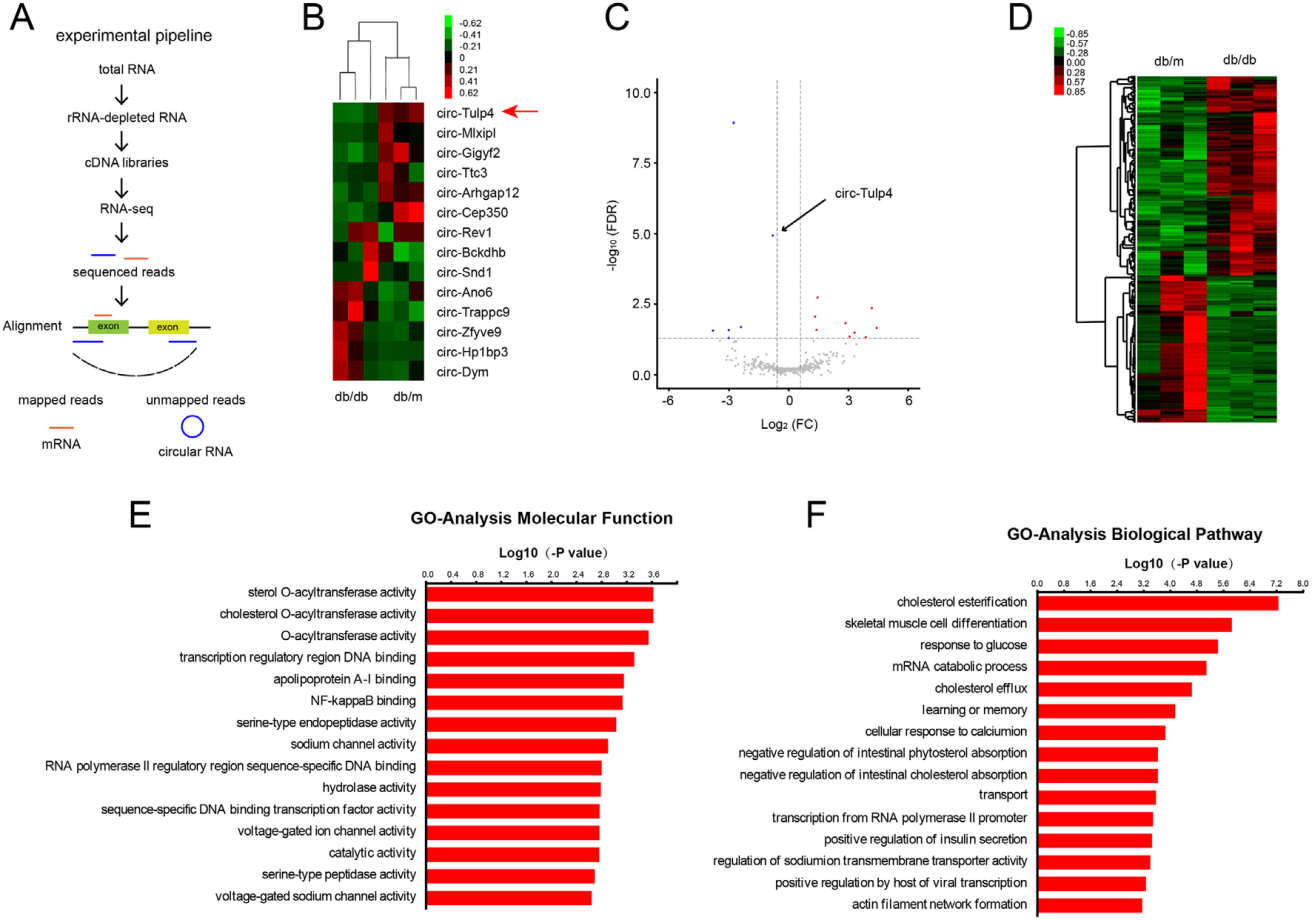
RNA-seq analysis of circRNA and mRNA expression patterns in islets of three db/db and three db/m mice. (A) RNA-seq experiment pipeline. (B) Clustered heat map of the differentially expressed circRNAs with a fold change of more than 1.5, which displayed on a scale from green (low) to red (high). (C) Volcano plot showing circular RNA log_2_ fold changes (FC) (on the X*-*axis) and the corresponding tempered log_10_ FDR values (on the Y*-*axis). (D) Profile of upregulated and downregulated mRNAs. Green indicated low expression and red high expression. The top 15 involved molecular functions (E) and biological pathways (F) in dysregulated mRNAs analyzed by gene ontology (GO).

To investigate the involvement of circRNAs in the regulation of β-cell dysfunction and the development of T2DM, the transcriptomes of isolated islets were compared using the Hiseq Sequencer platform. The characteristics of the animal models used in this study were presented in Supplementary Fig. 1. Differentially expressed circRNAs were identified by comparing db/db and db/m samples (10-week-old). The analysis detected about 5000 circRNAs expressed in mouse pancreatic islets, of which 346 circRNAs exhibited significant differences in expression, and 14 showed a significant difference with a fold change ≥ 1.5. In detail, eight genes were upregulated, whereas six downregulated. The 14 dysregulated circRNAs were plotted in Fig. 1A, B, and C. The chromosomal location and the fold changes of these circRNAs were shown in Supplementary Table 2. The expression of these circRNAs in primary islets were validated using specific divergent primers and Sanger sequencing. These results suggested that circRNAs were abundantly expressed in pancreatic islets, and various circRNA levels were dysregulated in diabetic mice models. Besides, the mRNA expression levels were compared, and the gene sets associated with cholesterol esterification were found to be the most significantly dysregulated (Fig. 1D, E and F). This suggested that cholesterol esterification might be important for β-cell functions under diabetic condition. However, the function of dysregulated circRNAs in β cells and its relationship with cholesterol esterification-related genes remained unknown.

### Circ-Tulp4 was highly expressed in mice pancreatic islets and affected by the lipotoxic condition

**Figure 2:**
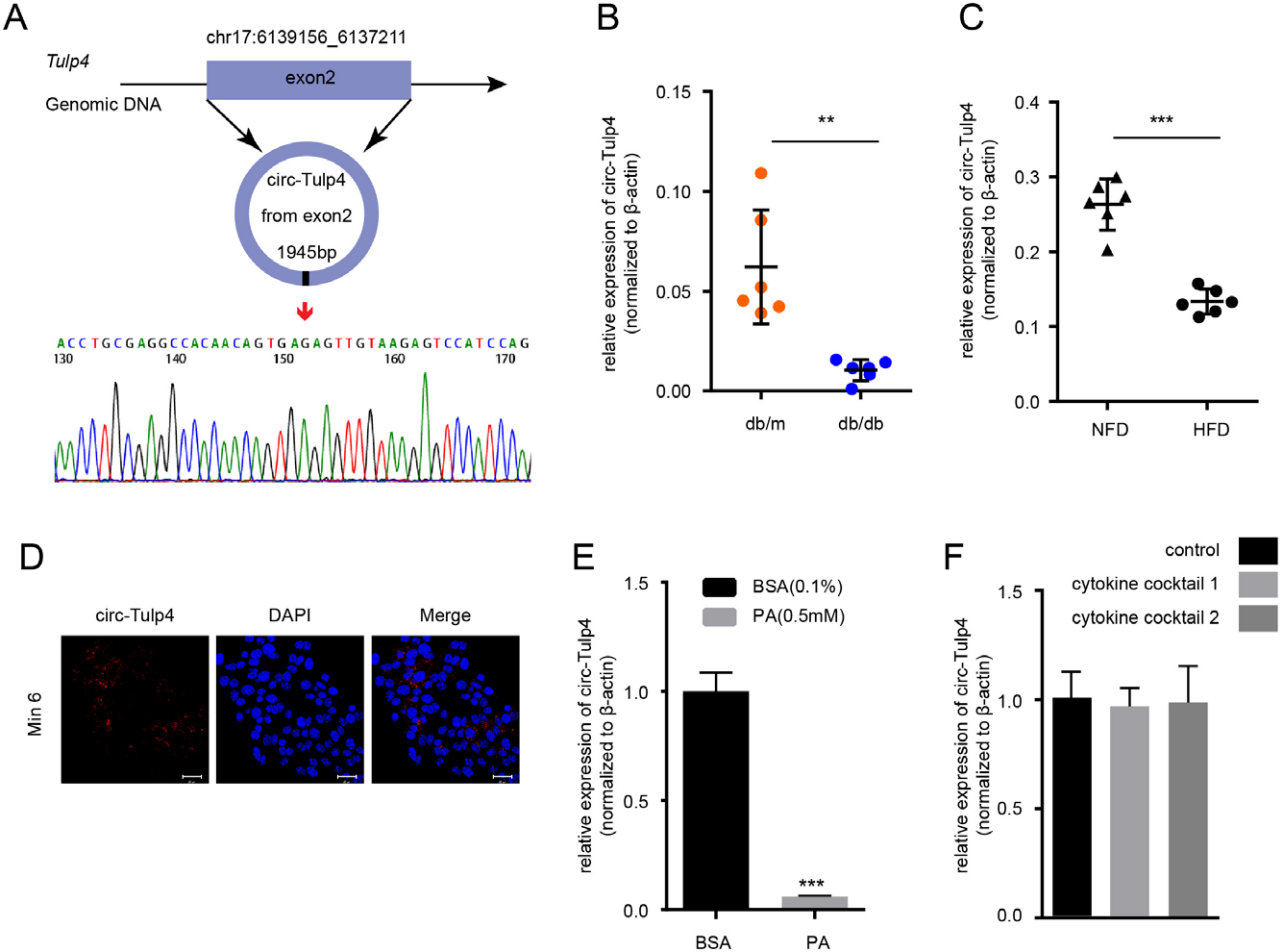
Expression of circ-Tulp4 decreased in islets of db/db mice and Min6 cells exposed to PA. (A) Schematic representation of circ-Tulp4 formation; the splice junction sequence was Sanger sequenced using cDNA samples from PCR. Expression level of circ-Tulp4 was detected by qRT-PCR in islets of db/db and db/m mice (B), or in C57BL/6J mice on a normal control (NFD) or high-fat diet (HFD) (C) (*n* = 6, respectively). **P < 0.01; ***P < 0.001 vs indicated groups. β-actin served as an internal control. (D) *In situ* hybridization (FISH) was used to determine the localization of circ-Tulp4 in Min6 cells. Expression level of circ-Tulp4 in Min6 cells exposed to PA (E) or cytokine cocktails (F). ***P < 0.001 vs the control group. The concentration of cytokine cocktails was depicted in detail in the Supplementary Materials and methods (see section on supplementary materials given at the end of this article) in detail.

Next, circ-Tulp4 was chosen for its displaying the largest number of reads in Supplementary Table 2, with 150.87 normalized reads and the expression decreased by 1.76-fold in the db/db mice. The genomic structure of circ-Tulp4 was shown in Fig. 2A; it comprised an exonic sequence of 1945 bp with high conservation across species (using MUSCLE tools shown in the Supplementary Materials and methods). We confirmed that levels of circ-Tulp4 were decreased in islets of db/db mice and HFD C57BL/6J mice (Fig. 2B and C). Localization in β cells was evaluated by *in situ* hybridization with probes covering the junction site of circ-Tulp4 (Fig. 2D).

The expression level in β cells exposed to elevated concentrations of non-esterified fatty acid (NEFA) was tested to determine whether lipotoxic condition contributed to the expression changes of circ-Tulp4 detected in diabetic mice. The exposure of Min6 cells to palmitate acid (PA) (0.5 mM) resulted in significantly lower expression levels of circ-Tulp4 (Fig. 2E). However, the circ-Tulp4 level was not significantly affected in Min6 cells treated with diabetes-associated cytokine cocktails (Fig. 2F). Thus, the analysis revealed that circ-Tulp4 was regulated by NEFA in Min6 cells, suggesting that the low expression of circ-Tulp4 might be related to β-cell lipotoxicity and the development of T2DM.

### Circ-Tulp4 maintained β-cell function under lipotoxic conditions

**Figure 3:**
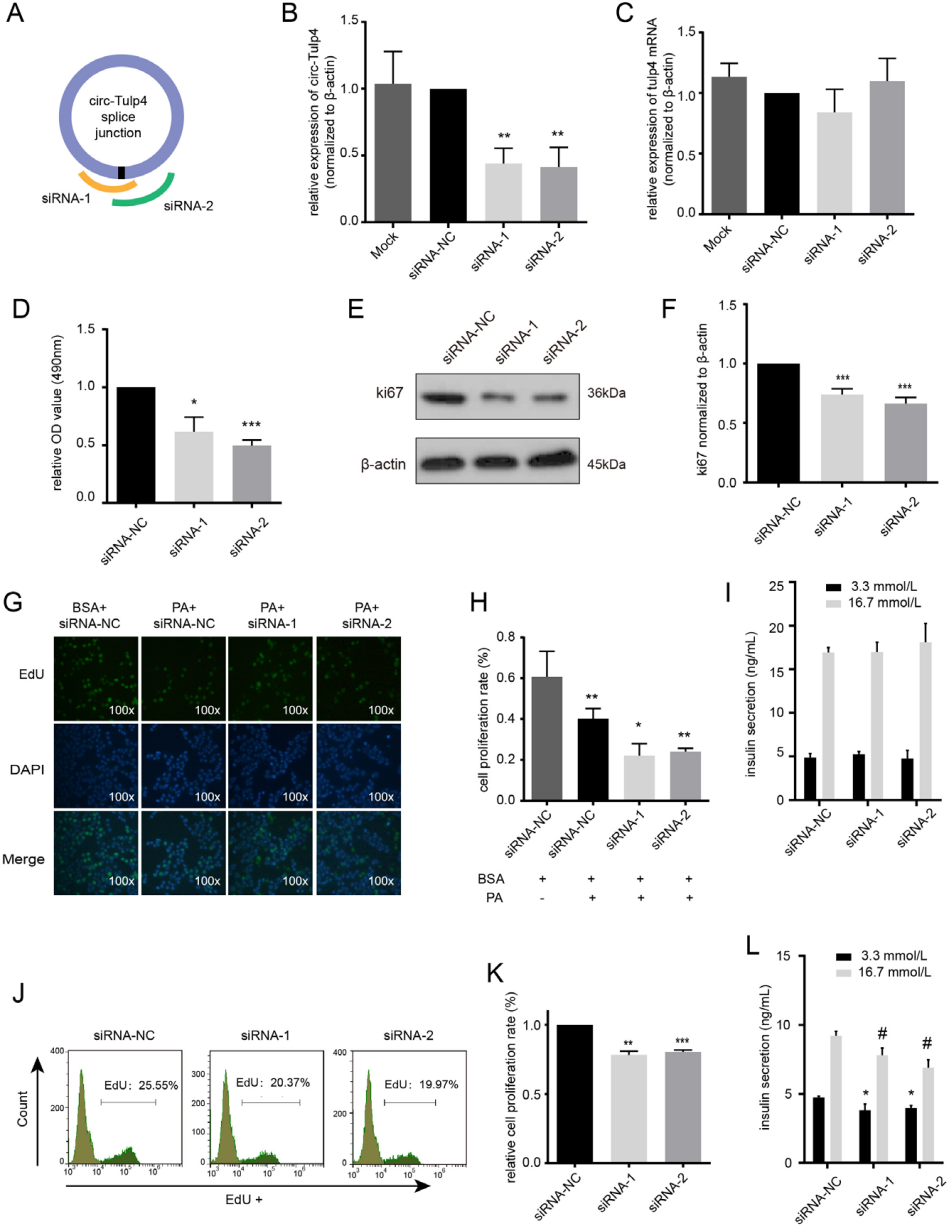
Downregulation of circ-Tulp4 inhibited β-cell function in the presence of PA. (A) Schematic representation of designed siRNAs for circ-Tulp4 at splice junction. Expression of circ-Tulp4 (B) and tulp4 mRNA (C) were detected by qRT-PCR in siRNA-1, siRNA-2, or mock-transfected Min6 cells. (D) The Min6 cells were transfected with circ-Tulp4 siRNA-1 or siRNA-2 for 48 h, followed by PA (0.5 mM) treatment for 24 h. Cell proliferation ability was detected by MTS. Western blot assays were used to analyze the expression level of ki67 in transfected cells (E and F). To assess the cell proliferation rate, EdU incorporation was detected by microscope (G and H) or flow cytometry (J and K). Insulin secretion levels of Min6 cells after BSA (I) or PA (L) treatment were analyzed using GSIS assay. Green: EdU-positive staining. Blue: Hoechst 33342 staining of the nuclei. *P < 0.05; **P < 0.01; ***P < 0.001 vs siRNA-NC transfected cells, or solvent (BSA) treated cells, or siRNA-NC transfected cells in 3.3 mmol/L glucose. ^#^P < 0.05 vs siRNA-NC transfected cells in 16.7 mmol/L glucose.

Circ-Tulp4 expression was modulated to establish the biological role in β cells. Two siRNAs or a negative control sequence (siRNA-NC) was transiently transfected into Min6 cells, and the effect of siRNA-mediated knockdown of circ-Tulp4 was assessed after 48 h. As expected, the treatment effectively silenced the circular transcripts for approximately 50% reduction, whereas it did not affect the expression of linear mRNAs (Fig. 3A, B and C). A subsequent MTS assay showed that cell survival significantly reduced in the circ-Tulp4 siRNA-treated group under lipotoxic condition, whereas no obvious difference was observed by downregulating circ-Tulp4 alone (Fig. 3D and Supplementary Fig. 2A, B, C). The cell proliferation rate consistently diminished by EdU assays (Fig. 3G, H, J, Kand Supplementary Fig. 2D, E); so was the proliferation-related protein level of ki67 (Fig. 3E, F and Supplementary Fig. 2F). Additionally, defective GSIS was observed after knocking down circ-Tulp4 under lipotoxic condition (Fig. 3I and L). In contrast, circ-Tulp4 silencing did not affect the apoptosis of Min6 cells (Supplementary Fig. 2I and L) or alter insulin biosynthesis (Supplementary Fig. 2G and H). Hence, the silencing of circ-Tulp4 negatively affected the β-cell function.

**Figure 4:**
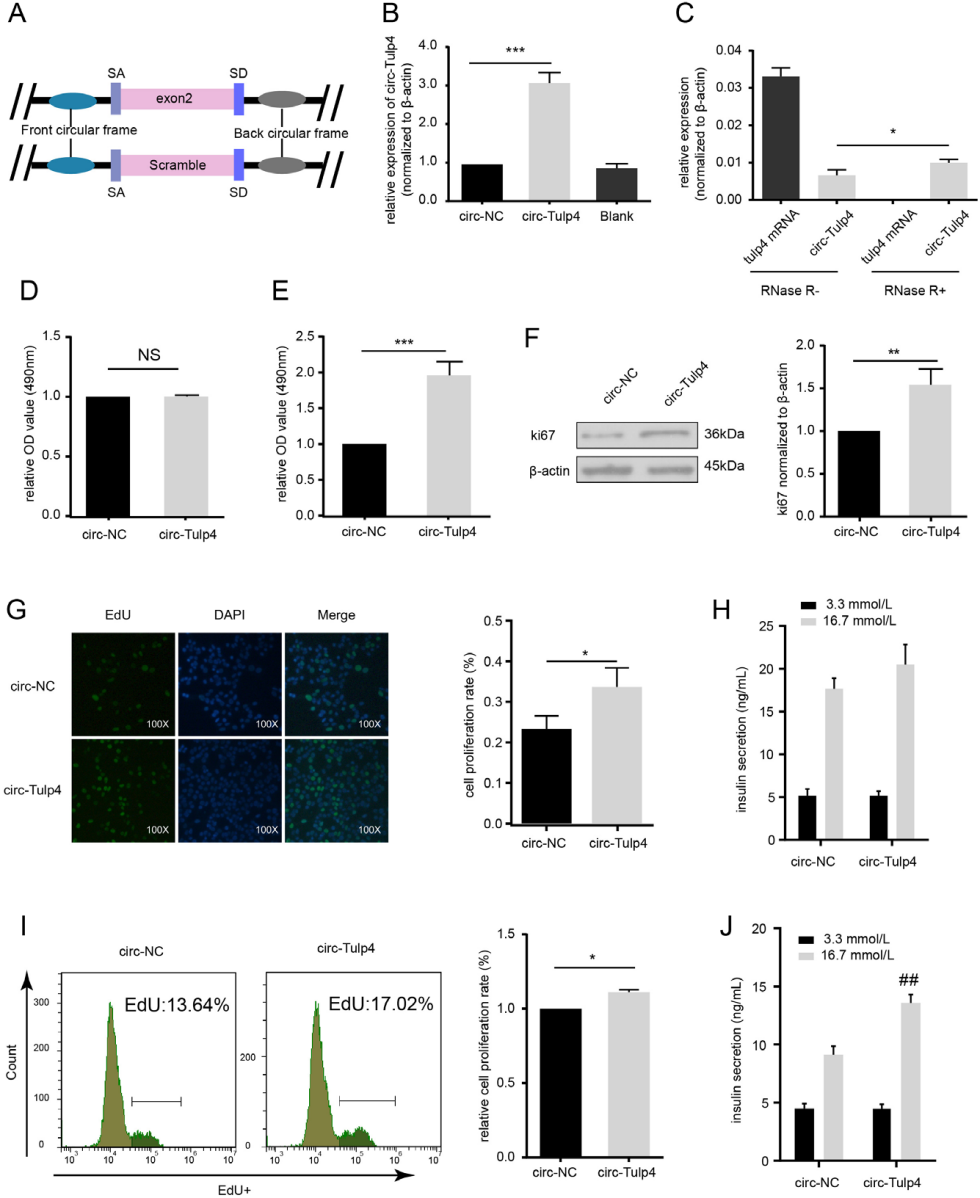
Upregulation of circ-Tulp4 alleviates β-cell dysfunction in the presence of PA. (A) Schematic representation of circ-Tulp4 and NC AAV vector construction. Expression of circ-Tulp4 and tulp4 mRNA was measured by qRT-PCR in the circ-Tulp4 or NC vector-infected Min6 cells (B), with or without RNase R treatment (C). Min6 cells were infected with indicated AAV vectors for 48 h, followed by BSA (D) or PA (E) treatment for 24 h. Cell proliferation ability was detected by MTS. (F) Western blot assays were used to analyze the protein expression level of ki67. To assess the cell proliferation rate, EdU incorporation was detected using a microscope (G) or flow cytometry (I). GSIS assay was used to detect insulin secretion of Min6 cells after BSA (H) or PA (J) treatment. Green: EdU-positive staining. Blue: Hoechst 33342 staining of the nuclei. *P < 0.05; **P < 0.01; ***P < 0.001 vs NC vector infected cells. ^##^*P* < 0.01 vs NC vector infected cells in 16.7 mmol/L glucose.

Next, whether the upregulation of circ-Tulp4 level affected β-cell function was also investigated. AAV vector overexpressing circ-Tulp4 could efficiently produce a circular transcript partly resistant to RNase R digestion (Fig. 4A, B and C). As shown in Fig. 4D and  E, PA inhibition of cell survival significantly decreased in Min6 cells with overexpression of circ-Tulp4. These effects were further confirmed with increased cell proliferation rate, as shown in the EdU assays (Fig. 4G and I) and increased protein level of ki67 (Fig. 4F). Additionally, though no obvious change in the mRNA levels of insulin1 (ins1) or insulin2 (ins2) was observed, insulin secretion was notably improved after overexpressing circ-Tulp4 (Fig. 4H, J and Supplementary Fig. 3C). Also, apoptosis rate was unchanged with the overexpression of circ-Tulp4 (Supplementary Fig. 3A and B). In all, the overexpression of circ-Tulp4 alleviates β-cell dysfunction under lipotoxic condition.

Altogether, these results showed that circ-Tulp4 played a vital role in regulating β-cell function under the lipotoxic environment, which may be a potential target in treating T2DM.

### Bioinformatics identifies target genes affected by circ-Tulp4 changes

**Figure 5:**
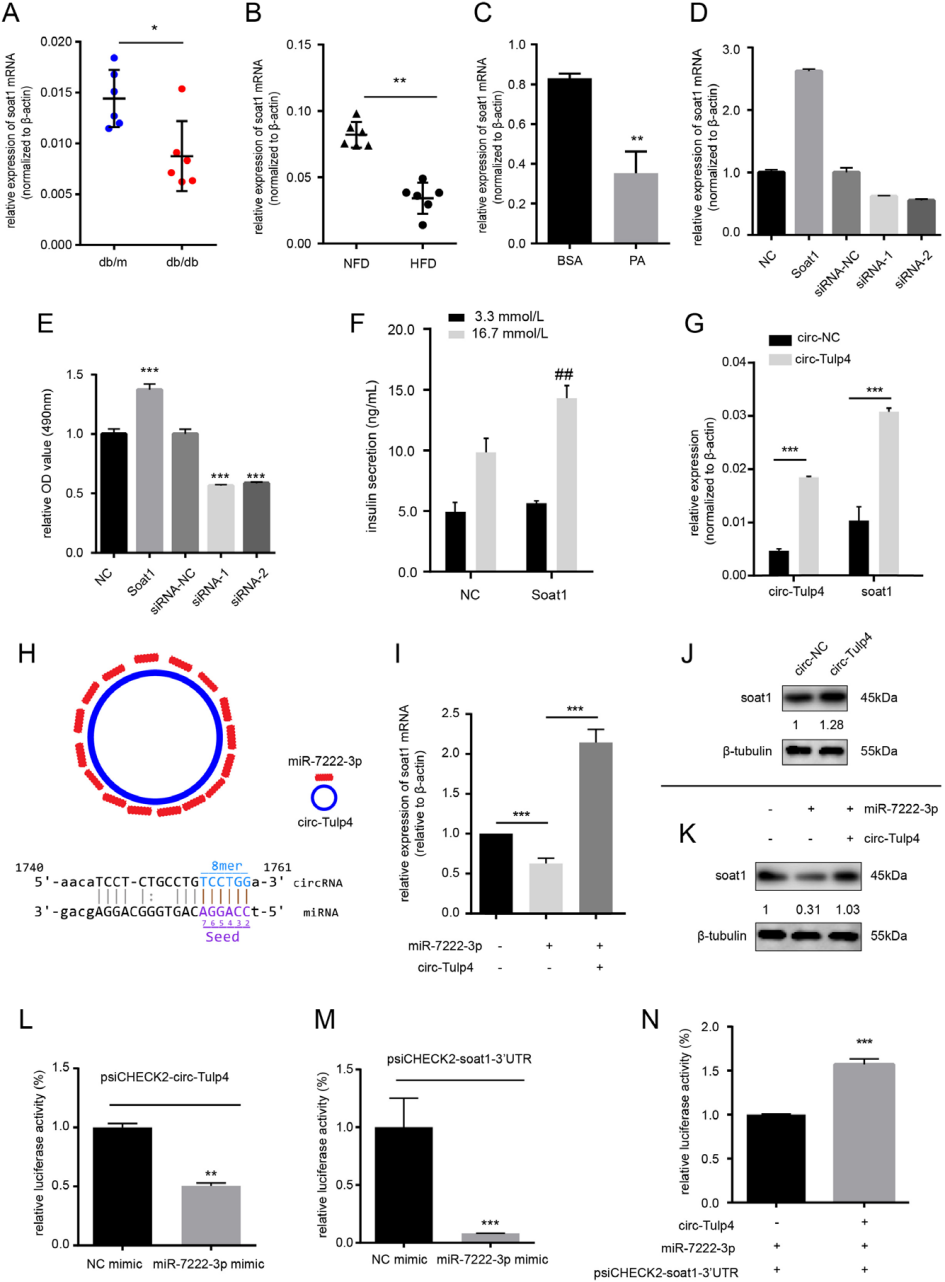
Expression of soat1 was downregulated in diabetic mice islets and Min6 cells exposed to PA, and it was regulated by circ-Tulp4 and miR-7222-3p. Expression level of soat1 in islets of db/db and db/m mice (A), or in C57BL/6J mice on a normal control (NFD) or high-fat diet (HFD) (B) (*n* = 6, respectively). *P < 0.05; **P < 0.01 vs indicated groups. β-actin served as an internal control. (C) Expression level of soat1 in Min6 cells exposed to PA. **P < 0.01 vs the solvent (BSA) control group. Soat1 was knocked down or overexpressed in Min6 cells, followed by PA (0.5 mM) treatment for 24 h (D). Cell proliferation ability was detected by MTS (E). Insulin secretion was detected by GSIS assay after overexpressing soat1. ^##^*P* < 0.01 *vs* NC vector treated cells in 16.7 mmol/L glucose (F). Relative expression of circ-Tulp4 and soat1 mRNA after circ-Tulp4 or NC vector infection (G). The 16 predicted binding sites for miR-7222-3p on circ-Tulp4 (H). Expression level of soat1 mRNA (I) or protein (K) in Min6 cells treated with either miR-7222-3p mimic or co-treated with miR-7222-3p mimic and circ-Tulp4 vector (I). Protein level of soat1 in Min6 cells after circ-Tulp4 vector infection (J). Dual-luciferase analysis in HEK293 cells after indicated treatment (L and M). Competitive luciferase analysis in HEK293 cells (N). **P < 0.01; ***P < 0.001 vs the indicated groups.

As described in Fig. 1D and F, cholesterol esterification might play an important role in β-cell function under diabetic condition. The biological analysis data showed that the mRNA level of sterol O-acyltransferase 1 (*soat1*), a key enzyme gene in cholesterol esterification, was lower in diabetic mice islets (*P* < 0.05). The expression level of soat1 mRNA was tested to validate the bioinformatics analysis experimentally. The results showed that the expression of soat1 significantly decreased in the db/db mice islets and HFD mice islets (Fig. 5A and B). *In vitro*, the expression of soat1 consistently displayed significantly decreased levels under lipotoxic condition (Fig. 5C). These findings suggested that soat1 might be related to β-cell dysfunction in the development of T2DM. Previous studies demonstrated that soat1 was needed for the cell proliferation and activation of adaptation to pathological concentrations of NEFA (Busch *et al.* 2005, Yue *et al.* 2014, Li *et al.* 2016, Stopsack *et al.* 2017). Hence, the next step was to test the hypothesis of whether soat1 affected β-cell function and whether circ-Tulp4 regulated the expression of soat1.

**Figure 6:**
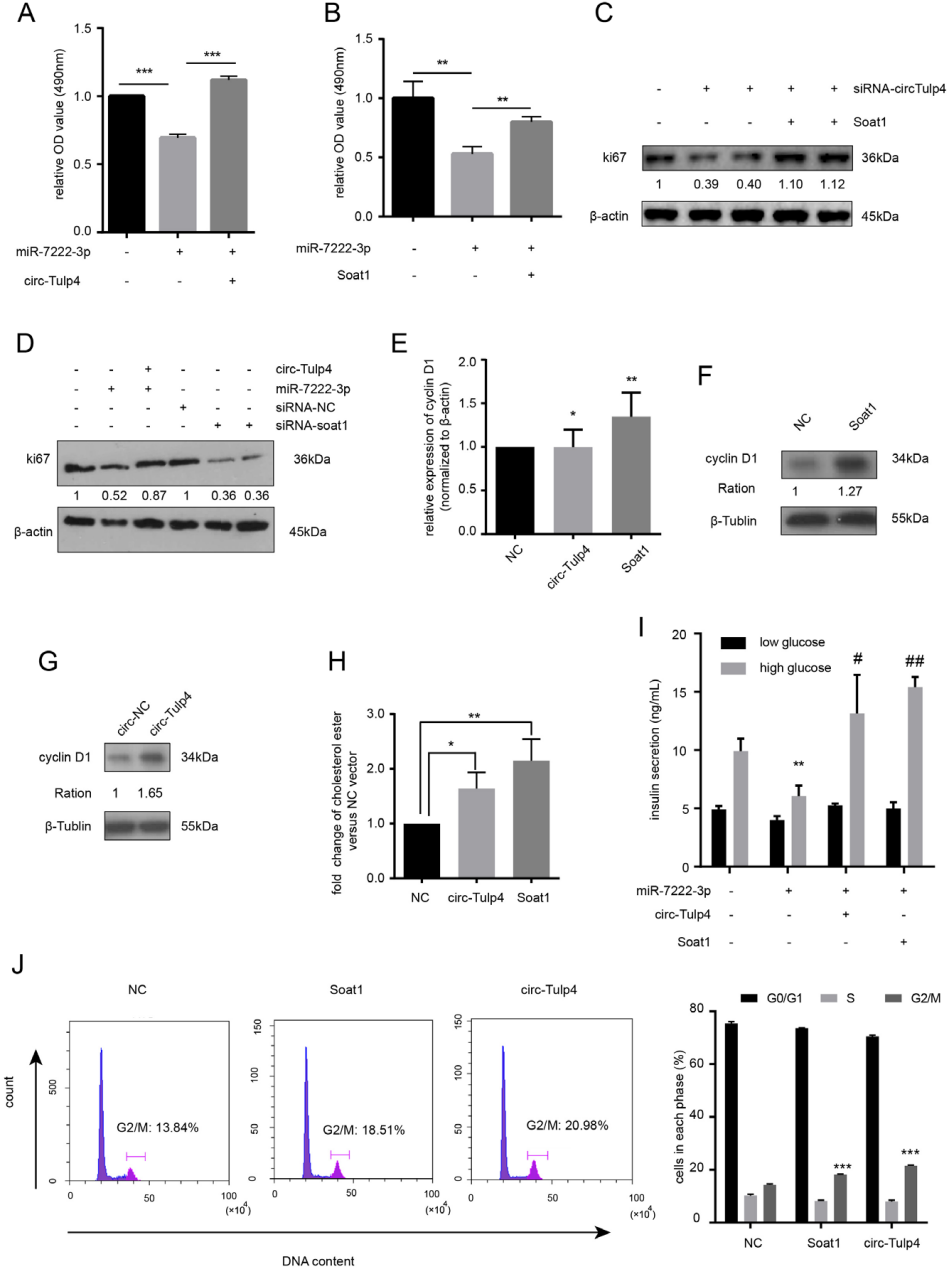
Upregulation of circ-Tulp4 affected the proliferation of β cells by sponging miR-7222-3p and regulating soat1/cyclin D1 signaling. Min6 cells were transfected with miR-7222-3p mimic, or co-treated with circ-Tulp4 vector (A) or Soat1 vector (B) for 48 h, followed by PA (0.5 mM) treatment for 24 h. Cell proliferation ability was detected by MTS. (C) Min6 cells were transfected with siRNA-1 or siRNA-2 for circ-Tulp4, or co-treated with Soat1 vector for 48 h, followed by PA (0.5 mM) treatment for 24 h. Western blot assays were used to analyze the protein expression level of ki67. (D) Min6 cells were transfected with miR-7222-3p mimic, or co-treated with circ-Tulp4 vector for 48 h, followed by PA (0.5 mM) treatment for 24 h. Min6 cells were transfected with siRNA-1 or siRNA-2 for soat1 for 48 h, followed by PA (0.5 mM) treatment for 24 h. Protein expression of ki67 in indicated groups. The expression level of cyclin D1 mRNA (E) or cyclin D1 protein (F and G) in Min6 cells infected with circ-Tulp4 vector or Soat1 vector. Cell cholesterol easter content was examined after overexpressing circ-Tulp4 or Soat1 (H). GISI assay was performed to examine the insulin secretion of Min6 cells transfected with miR-7222-3p mimic, or co-treated with circ-Tulp4 vector or Soat1 vector (I). **P < 0.01 vs the NC treated group in 16.7 mmol/L glucose. ^#^*P* < 0.05; ^##^ *P* < 0.01 vs the miR-7222-3p mimic treated group in 16.7 mmol/L glucose. Cell cycle distribution was investigated by flow cytometry in Min6 cells infected with circ-Tulp4 vector or Soat1 vector (J). *P < 0.05; **P < 0.01; ***P < 0.001 vs the indicated groups.

Our previous data showed that silencing of soat1 impaired β-cell survival under lipotoxic condition, whereas the upregulation of soat1 had the opposite effect (Fig. 5E and Supplementary Fig. 3E, F). Protein level of ki67 was reduced after knocking down soat1 expression under lipotoxic condition (Fig. 6D and Supplementary Fig. 4E). Infecting Min6 cells with soat1 overexpression vector could elevated insulin secretion in 16.7 mmol/L glucose (Fig. 5F). Furthermore, the ectopic expression of circ-Tulp4 significantly promoted soat1 mRNA (Fig. 5G) and protein expression in Min6 cells (Fig. 5J). Hence, soat1 was found to be related to β-cell dysfunction and post-transcriptionally modulated by circ-Tulp4. However, the mechanism remained to be further elucidated.

We postulated that circ-Tulp4 might act as a microRNA sponge to regulate the expression of soat1. To test this speculation, miRanda (http://www.microrna.org/microrna/home.do) and RNAhybrid (http://bibiserv.techfak.uni-bielefeld.de/rnahybrid/) were used to predict candidate miRNA, and miRNA with more than 10 potential binding sites on circ-Tulp4 were selected. Among these, only miR-7222-3p was able to reduce soat1 mRNA (Fig. 5H and  I) and protein (Fig. 5K) expression levels, and the effect could be rescued via co-overexpressing circ-Tulp4 (Supplementary Fig. 3G, H and I). Next, to validate whether miR-7222-3p could interact with circ-Tulp4 and soat1 3’-UTR, a luciferase reporter assay was performed. The reporter construct and miR-7222-3p mimic or NC mimic were co-transfected into the HEK293 cells. The luciferase activity assay showed that miR-7222-3p bound to circ-Tulp4 and soat1 3’-UTR directly (Fig. 5L and M). Moreover, a competitive luciferase assay was performed to test the effect of circ-Tulp4 on miR-7222-3p activity. In the HEK293 cells, the overexpression of circ-Tulp4 blunted the negative effect of miR-7222-3p on the luciferase expression of luciferase reporter containing soat1 3’-UTR, whereas the NC construct did not (Fig. 5N). Taken together, these results demonstrated that circ-Tulp4 functioned as a miR-7222-3p sponge in β cells.

Finally, whether the capacity of circ-Tulp4 to maintain β-cell function under NEFA-induced lipotoxicity depended on miR-7222-3p inhibition was investigated. The overexpression of miR-7222-3p receded cell proliferation, which was enhanced following the co-overexpression of circ-Tulp4 (Fig. 6A and D). As soat1 is the target gene of miR-7222-3p, the present study further revealed that the ectopic expression of miR-7222-3p inhibited the survival of Min6 cells, while the co-overexpression of soat1 re-enhanced cell survival (Fig. 6B). Cells with inhibited circ-Tulp4 expression showed lower ki67 protein levels compared with the control group; while the co-overexpression of soat1 recapitulated the effects of circ-Tulp4 activation (Fig. 6C). On the other hand, no significant differences in cell proliferation were observed under basal condition (Supplementary Fig. 4A, B, C and D). Insulin secretion was significantly decreased after ectopic expression of miR-7222-3p, while improved following the co-expression of circ-Tulp4 or soat1 (Fig. 6I). Besides, the ectopic expression of miR-7222-3p did not affect cell apoptosis either in basal or lipotoxic condition, and co-overexpression of miR-7222-3p and circ-Tulp4 or soat1 also displayed negative results (Supplementary Fig. 4I). These data suggested that the effect of circ-Tulp4 on β-cell function related to an increased expression of soat1 through the inhibition of miR-7222-3p activity under lipotoxic condition.

### Soat1 enhanced cell proliferation through cyclin D1 activation

This study demonstrated that miR-7222-3p suppressed cell survival in the presence of PA, and the upregulation of circ-Tulp4 and soat1 rescued miR-7222-3p inhibition on cell survival. Previous studies indicated that cyclin D1, which positively regulated the cell cycle, was a potential soat1 target (Batetta *et al.* 2003). However, the effect of soat1 upregulation on the cell cycle was still not verified in β cells. As revealed by qRT-PCR and western blot analysis, cyclin D1 mRNA (Fig. 6E) and protein expression levels (Fig. 6F) were modified by the upregulation of soat1. As soat1 was post-transcriptionally regulated by circ-Tulp4, the expression level of cyclin D1 was also increased on the overexpression of circ-Tulp4 (Fig. 6G and H). Under the basal condition, the expression of cyclin D1 was unchanged upon upregulation of circ-Tulp4 or soat1 (Supplementary Fig. 4F, G and H). As shown by flow cytometry assays (Fig. 6J), the increased expression of soat1 displayed beneficial effects on β-cell proliferation, and the overexpression of circ-Tulp4 also increased the percentage of cells in the G2/M phase of the cell cycle, promoting proliferation. The level of cholesterol ester, the product of soat1 catalyzation, was also increased upon upregulation of circ-Tulp4 or soat1 in Min6 cells (Fig. 6H). Hence, the translational upregulation of soat1 and cyclin D1 could explain the phenotypic traits of β cells overexpressing circ-Tulp4.

## Discussion

β-Cell lipotoxicity and progressive dysfunction are predisposing factors for T2DM. Chronic exposure to elevated NEFA is detrimental for β cells, resulting in reduced proliferation and increased apoptosis. A persistent imbalance between excessive β-cell apoptosis and restricted proliferation leads to β-cell mass loss and reduced insulin secretion (Biden *et al.* 2014). Thus, controlling lipid-derived β-cell adaptive transitions is indispensable for maintaining glucose homeostasis. Notwithstanding its importance, β-cell adaptive regulatory networks and molecular mechanisms are still undefined, particularly for circRNAs. This study proved that the high abundance of circ-Tulp4 in β cells inhibited miR-7222-3p mediated suppression of soat1 expression, reinforcing cyclin D1 expression and cell cycle progression, and alleviating β-cell dysfunction under lipotoxic condition.

Recently, a large number of circRNAs have been revealed with novel bioinformatic approaches and been detected in mammalian cells. Nevertheless, only a handful of them is functionally characterized. In this study, more than 5000 circRNAs were detected using RNA sequencing, indicating that circular transcripts were abundant in islets. Among these, 346 displayed dysregulated expression in diabetic mice, and 14 previously annotated circRNAs displayed different expression patterns with more than 1.5-fold change. A similar expression pattern in circ-Arhgap12 was also observed by Stoll *et al.* (2018) previously, analyzing diabetic mice islets circRNAs expression by microarray. Besides, we found that silencing circ-Arhgap12 didn’t affect β-cell proliferation, which was consistent with Stoll’s study (data was not shown) (Stoll *et al.* 2018). In this study, circ-Tulp4 was selected for its upmost number of reads among candidate circRNAs. Both the human circ-Tulp4 and mouse circ-Tulp4 were transcribed from the second exon of TUB like protein 4 (*TULP4*) gene. The sequences of circ-Tulp4 were well conserved between mouse and human, and it was also found expressed in mouse brain (Supplementary Materials and methods) (Rybak-Wolf *et al.* 2015). However, the functional role was not tested before. Here, we found that circ-Tulp4 expression was reduced in diabetic mice islets and Min6 cells exposed to NEFA, reflecting an important role in the regulation of β-cell function. Additionally, we tested the second significantly downregulated gene of circ-Mlxipl. We found that it was also regulated by NEFA (data in submission). The functional role of other differentially expressed circRNAs might affect proliferation, apoptosis, or insulin secretion; however, it remained to be defined.

Previous studies explore the involvement of circRNAs in cell proliferation through various molecular mechanisms (Rybak-Wolf *et al.* 2015). For example, circHIPK3 promoted proliferation of human tumor cells by binding with multiple miRNA, and a research team showed that circ-SHPRH, circ-FBXW7, and circ-PINT suppressed human glioblastoma growth through direct protein translation (Zheng *et al.* 2016, Yang *et al.* 2018, Zhang *et al.* 2018*a*). The studies by Du *et al.* (2016) showed that circFoxo3 might bind and sequester cell cycle-related proteins outside the nucleus to inhibit cell proliferation. Our study demonstrated a new pathway regarding the positive association between cell proliferation and circ-Tulp4 in β cells, which interacted and modulated the activity of miR-7222-3p. Besides, increased insulin secretion was also observed upon overexpression of circ-Tulp4. These data collectively illustrated that circ-Tulp4 was significant in regulating β-cell function, which might be developed as a therapeutic target for T2DM.

Increasing evidence suggested that the function of circRNA is associated with its subcellular compartmentalization. Considering the enrichment of circ-Tulp4 in the cytoplasm, it was speculated that it might act as a sponge by sequestering miRNA to promote the translation of soat1 mRNA. Using bioinformatic approaches, circ-Tulp4 was predicted to target numerous miRNA. Among these, only miR-7222-3p was found to have an impact on the expression of soat1. Previously, miR-7222-3p was found expressed in mouse liver tissues (http://mirtarbase.mbc.nctu.edu.tw/) (Luna *et al.* 2017). Here, we found that miR-7222-3p was enriched in Min6 cells. Besides, since the human circ-Tulp4 was also comprised of exon, we hypothesized that it might also locate in the cytoplasm and could reserve β-cell function through targeting micro RNA. But due to the limited information regarding human β-cell mircoRNA expression (Cebola & Pasquali 2016), the detailed mechanism needs to be further detected. In our study, we found that miR-7222-3p could directly target circ-Tulp4 and repress expression of cholesterol esterification–related gene soat1. So far, these results highlighted some important mechanisms leading to lipotoxicity in β cells with reduced level of circ-Tulp4.

It is reported that the effect of specific fatty acids on β cells is directly related to the degree of their saturation (Janikiewicz *et al.* 2015, Liu *et al.* 2019). According to the Prospective Metabolism and Islet Cell Evaluation (PROMISE) survey, an impaired β-cell function and a higher risk of T2DM were correlated with the serum saturate lipid levels (Johnston *et al.* 2016). Besides, chronic exposure of β cells to unsaturated fatty acids significantly increases β-cell survival (Liu *et al.* 2019). Additionally, ** Busch *et al.* (2005) reported that the level of stearoyl-CoA desaturase (*SCD*), an important gene in the regulation of cholesterol unsaturation, was increased in PA-resistant Min6 cells. The inhibition of SCD reduced β-cell survival under lipotoxic condition. In the present study, gene sets involved in the regulation of cholesterol esterification, a key procedure in cholesterol unsaturation, were the most significantly dysregulated. Moreover, the level of soat1, a key enzyme during cholesterol esterification, was significantly lower in the diabetic mice islets and Min6 cells in the presence of PA. Given these findings, we hypothesized that β-cell failure due to chronic exposure to NEFA could be linked to decreased desaturation of fatty acids, which was achieved by cholesterol esterification.

Exogenous cholesterol spillover has two categories inside cells: cholesterol derivatives such as secondary messengers and toxic fatty acids (Chang *et al.* 2006). Increasing intracellular saturated fatty acids accumulation aggressively impair cell survival, inducing chronic toxicity to cells. Once exogenous cholesterol is incorporated into cholesterol ester (CE), the cells could take advantage of the esterified form to avoid the toxicity of excess saturated cholesterol. Indeed, CE is found to be a strong proliferative factor for cells, including cancer cells, stem cells, and retinal cells (Yue *et al.* 2014, Lee *et al.* 2015, Geng *et al.* 2016, Li *et al.* 2016). CE depletion by abrogating soat1 activity significantly hinders cell growth due to the elevation of saturated cholesterol levels (Buhman *et al.* 2000, Busch *et al.* 2005, Kedi *et al.* 2009, Li *et al.* 2016). Additionally, CE synthesis is essential for adaptation to increased cholesterol metabolic needs and intracellular cholesterol homeostasis (Busch *et al.* 2005, Lee *et al.* 2015). A general decrease in cholesterol esterification, particularly concerning the toxic PA, could impair the function and survival of β cells (Busch *et al.* 2005).

The terminal step of CE synthesis is soat1 catalyzation. Soat1 is ubiquitously expressed in tissues and highly conserved among mammals (Chang *et al.* 2006). The mRNA expression of soat1 is transcriptionally regulated through mechanisms involving extracellular cholesterol overloading and increasing metabolic demands. As mentioned earlier, the overexpression of soat1 promotes cell proliferation, and vice versa (Yue *et al.* 2014, Geng *et al.* 2016, Li *et al.* 2016, Stopsack *et al.* 2017), which might be associated with increasing cyclin D1 expression (Batetta *et al.* 2003). The overexpression of soat1 enhances 7-ketocholesterol (7KCh)-fatty acid ester formation from toxic 7KCh, which significantly protects cells from 7KCh-induced cell death (Lee *et al.* 2015). Our study also proved that the soat1 enhanced β-cell proliferation under PA-induced toxic condition, possibly through regulating cyclin D1 expression. Moreover, the impairment in insulin secretion was improved by upregulating soat1 expression. These findings imply that soat1 plays a key role in the adaptation to saturate fatty acids-induced cell impairment through various mechanisms.

Recently, soat1 is assumed to be a target for developing anti-atherosclerotic drugs. But neither overall nor myeloid-specific *SOAT1^−/−^* knockout mice exhibits a hyperglycemic condition (Lee *et al.* 2015). The reason might be correlated with the complex interactions of various systems in mice models. To understand the functional role of soat1 in β cells, β-cell-specific *SOAT1^−/−^* knockout mice is needed for further study. Besides, it is proposed that soat1 inhibitor could be used for developing anti-tumor drugs. The negative effect of soat1 inhibitors on pancreatic β cells should be noted, especially for patients with hyperlipidemia (Cheng *et al.* 2018). In diabetic mice, soat1 was significantly downregulated in islets. *In vitro*, it was proved that the expression of soat1 was epigenetically regulated by circ-Tulp4 and miR-7222-3p. Besides, CE content was increased in Min6 cells upon upregulation of soat1 or circ-Tulp4. Nevertheless, little is known about the mechanisms that trigger the transition of unsaturated fatty acids in β cells, and whether inhibition of CE storage or synthesis has negative effects on β-cell proliferation needs further confirmation.

Our study improved the understanding of the role of cholesterol esterification in β cells and opened opportunities for treating β-cell lipotoxic impairment. We confirmed that NEFA-induced β-cell impairment triggered the downregulation of circ-Tulp4, and circ-Tulp4 promoted cell function by inducing the expression of soat1 through the inhibition of miR-7222-3p. These results suggested that the upregulation of circ-Tulp4 might be a potential therapeutic intervention for T2DM. Besides, soat1 might be important for maintaining β-cell function under lipotoxic condition.

## Supplementary Material

Supplementary Fig. 1 Determination of body weight, blood glucose, food intake and glucose tolerance in db/db mice and db/m mice (A-D), or in C57BL/6J mice on a normal control or high-fat diet (F-H). Bodyweight (A), food intake (g/day/body weight) (B), and random blood glucose measured using a glucometer (Roche) from 6 to 9 weeks of age (n≥10 in A and C, n=5 in B). D Intraperitoneal glucose tolerance testing at 10 weeks of age. The mice were fasted overnight, and the blood glucose levels were monitored in response to 2 g/kg glucose (n=5). Blood glucose levels at all time points were comparatively high in db/db mice versus db/m mice. Data represent mean ± standard error of the mean. ***, P < 0.001 versus db/m mice. E Representative images of freshly isolated mice islets and insulin staining images. Insulin immunofluorescence assay was performed to confirm that the cells used for RNA-seq were acinar-free islets. The results indicated that isolated cells were mostly stained positive. Plots of body weight (F) and fasting blood glucose (G) of C57BL/6J mice over time (n≥10). A plot of time-dependent glucose tolerance curves in 37-week old C57BL/6J mice on a normal control (NFD) or high-fat diet (HFD) (n≥10). Blood glucose levels at all time points were comparatively high in HFD mice versus NFD mice. ***, P < 0.001 versus C57BL/6J mice on a NFD.

Supplementary Fig. 2 Min6 cells were transfected with circ-Tulp4 siRNAs for 24 h (A and C) or 48 h (B), followed by PA (0.5mM) (C) or solvent (BSA) treatment for 24 h (A and B). Cell proliferation ability was detected by MTS under basal condition or lipotoxic condition. To examine cell proliferation under basal condition, EdU assay (D and E) or western blot (F) was performed. Insulin biosynthesis (G-H) and apoptosis (I-L) were not affected by the silencing of circ-Tulp4. The protein expression level of cleaved caspase-3 was analyzed by Western blot under lipotoxic condition. (I and J). Min6 cells stained with Annexin V and propidium iodide (PI) were analyzed by flow cytometry for cell apoptosis assessment under basal (K) or lipotoxic (L) condition. *, P < 0.05 versus indicated groups.

Supplementary Fig. 3 To assess cell apoptosis, Min6 cells stained with Annexin V and propidium iodide (PI) were analyzed by flow cytometry (A-B). Expression of insulin1 mRNA (ins1) or insulin2 mRNA (ins2) was analyzed by qRT-PCR under lipotoxic condition after upregulating circ-Tulp4 (C) or soat1 (D) expression. Cell survival was examined by MTS in the siRNA-soat1 transfected cells (E) or Soat1 vector-infected cells (F) under basal condition. MiR-298-5p, miR-3113-3p, and miR-7222-3p demonstrated a potentially relevant role in regulating the expression of soat1, and verification of these microRNAs expressions in Min6 cells was shown (G). MiR-3113-5p served as a control. Expression level of soat1 in Min6 cells treated with either miR-298-5p mimic or co-treated with miR-298-5p mimic and circ-Tulp4 vector (H). Expression level of soat1 in Min6 cells treated with either miR-3113-3p mimic or co-treated with miR-3113-3p mimic and circ-Tulp4 vector (I). NS, Non-significance of difference. *, P < 0.05; **, P < 0.01 versus the indicated groups.

Supplementary Fig. 4 Min6 cells were transfected with miR-7222-3p mimic, or co-treated with circ-Tulp4 vector (A) or Soat1 vector (B) for 48 h, followed by BSA treatment for 24 h. Cell proliferation ability was detected by MTS. Min6 cells were transfected with miR-7222-3p mimic, or co-treated with circ-Tulp4 vector for 48 h(C); or transfected with siRNA-1 or siRNA-2 for circ-Tulp4, or co-treated with Soat1 vector for 48 h (D); or transfected with siRNA-1 or siRNA-2 for soat1 for 48 h (E), followed by BSA treatment for 24 h. Western blot assays were used to analyze the protein expression level of ki67. The expression level of cyclin D1 mRNA (F and G) or protein (H) in Min6 cells infected with circ-Tulp4 or Soat1 vector was analyzed. For apoptosis assessment, TUNEL staining was performed and TUNEL positive Min6 cells with indicated treatment were counted (I). Scale bar = 50 μm. Non-significant differences were observed in the above groups.

Supplementary Table 1 primers used for qRT-PCR in this study.

Supplementary Materials and methods

## Declaration of interest

The authors declare that there is no conflict of interest that could be perceived as prejudicing the impartiality of the research reported.

## Funding

This work was supported by the Department of Finance of Guangdong Province (Grant number: 20160902).

## Data availability statement

The data used to support the findings of this study are available from the corresponding authors on reasonable request.
