# Supplementary figures and images for: Circ-Tulp4 promotes β-cell adaptation to lipotoxicity by regulating soat1 expression
Source: J Mol Endocrinol. 2020 Sep 11;65(4):149–61. doi: 10.1530/JME-20-0079 (PMC7576671; doi:10.1530/JME-20-0079)

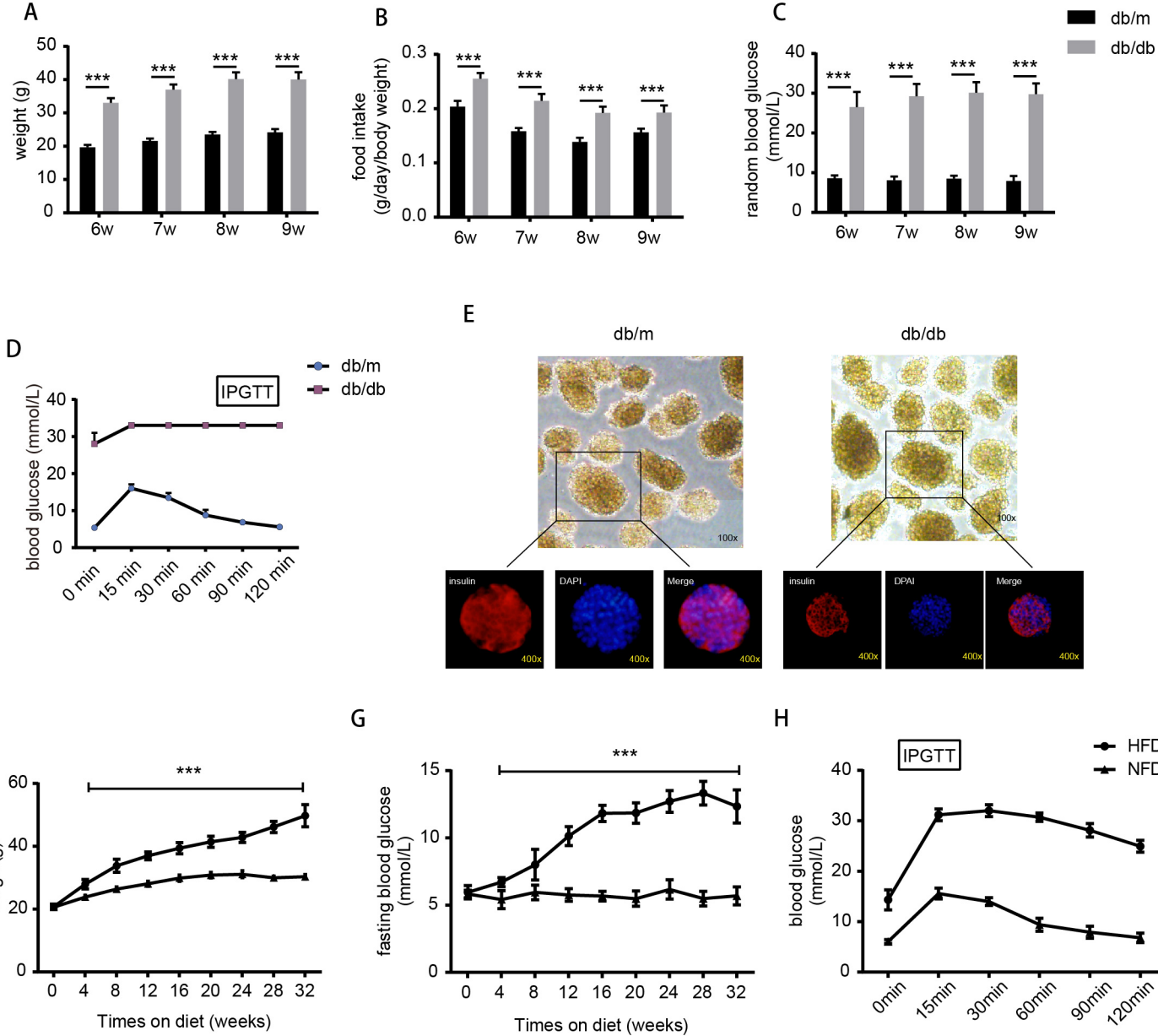

Supplement: Supplementary Fig. 1 Determination of body weight, blood glucose, food intake and glucose tolerance in db/db mice and db/m mice (A-D), or in C57BL/6J mice on a normal control or high-fat diet (F-H). Bodyweight (A), food intake (g/day/body weight) (B), and random blood glucose measured using a glucom [file supplementary_figure_1.pdf]

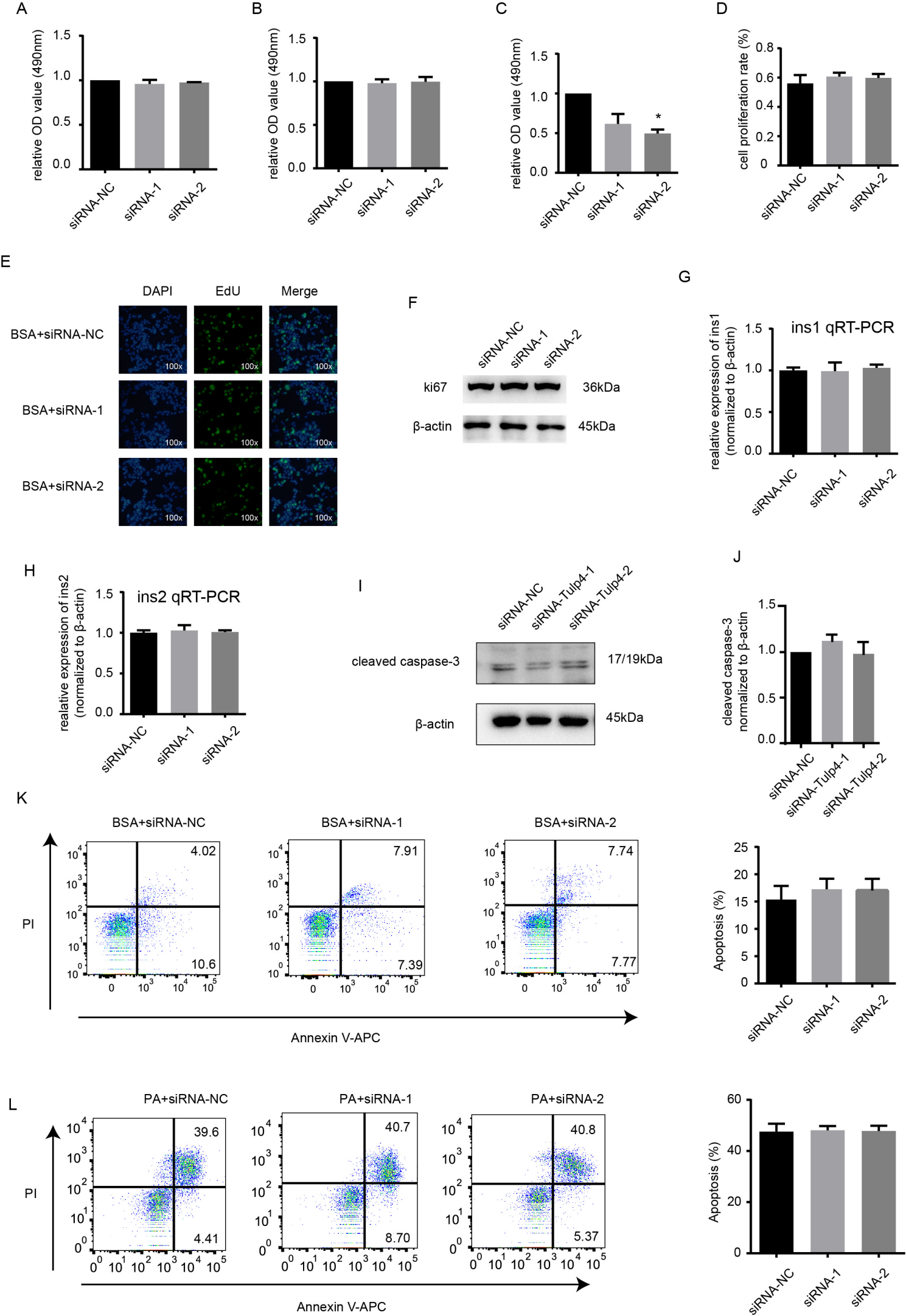

Supplement: Supplementary Fig. 2 Min6 cells were transfected with circ-Tulp4 siRNAs for 24 h (A and C) or 48 h (B), followed by PA (0.5mM) (C) or solvent (BSA) treatment for 24 h (A and B). Cell proliferation ability was detected by MTS under basal condition or lipotoxic condition. To examine cell proliferation [file supplementary_figure_2.pdf]

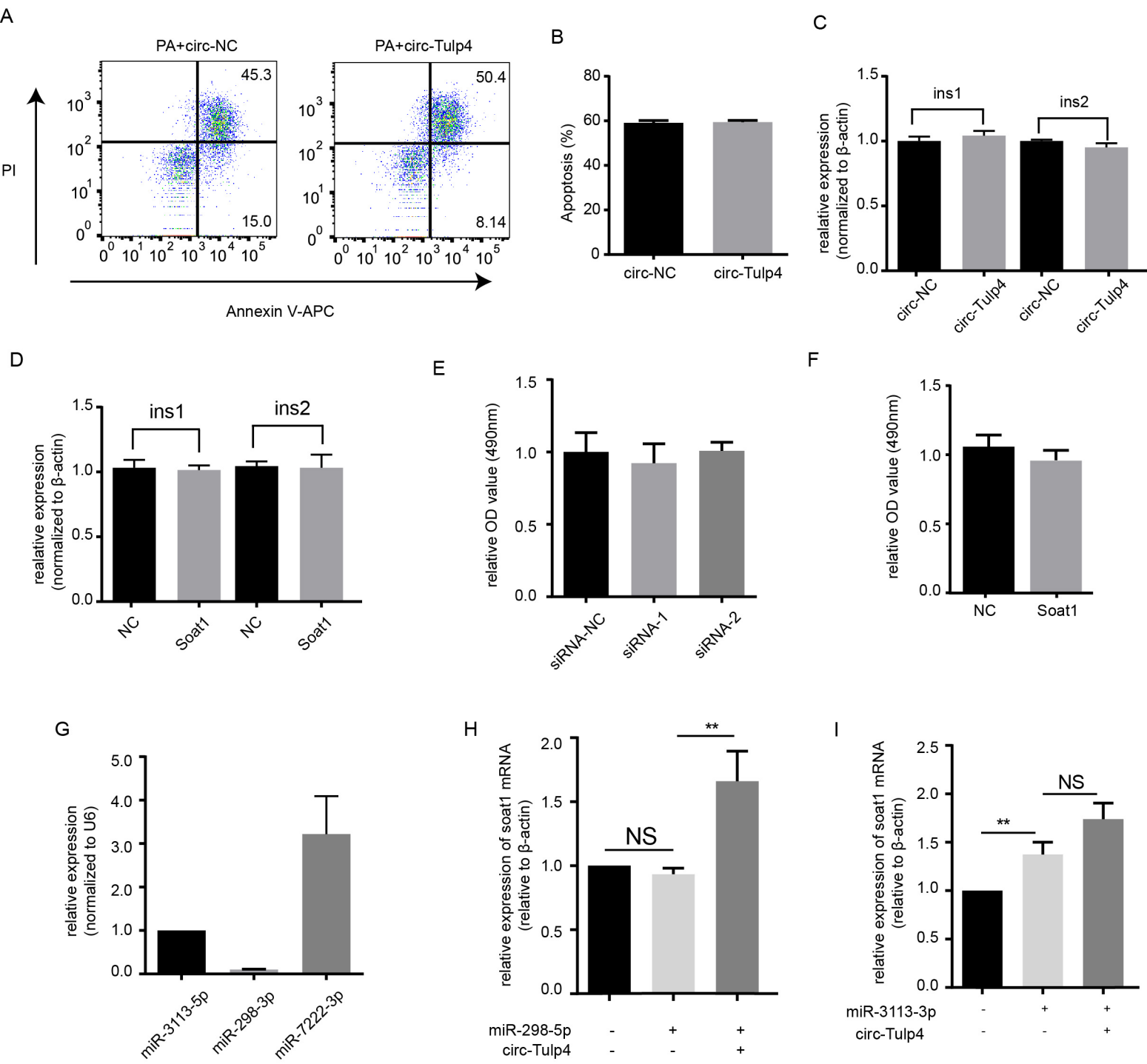

Supplement: Supplementary Fig. 3 To assess cell apoptosis, Min6 cells stained with Annexin V and propidium iodide (PI) were analyzed by flow cytometry (A-B). Expression of insulin1 mRNA (ins1) or insulin2 mRNA (ins2) was analyzed by qRT-PCR under lipotoxic condition after upregulating circ-Tulp4 (C) or soat1 (D [file supplementary_figure_3.pdf]

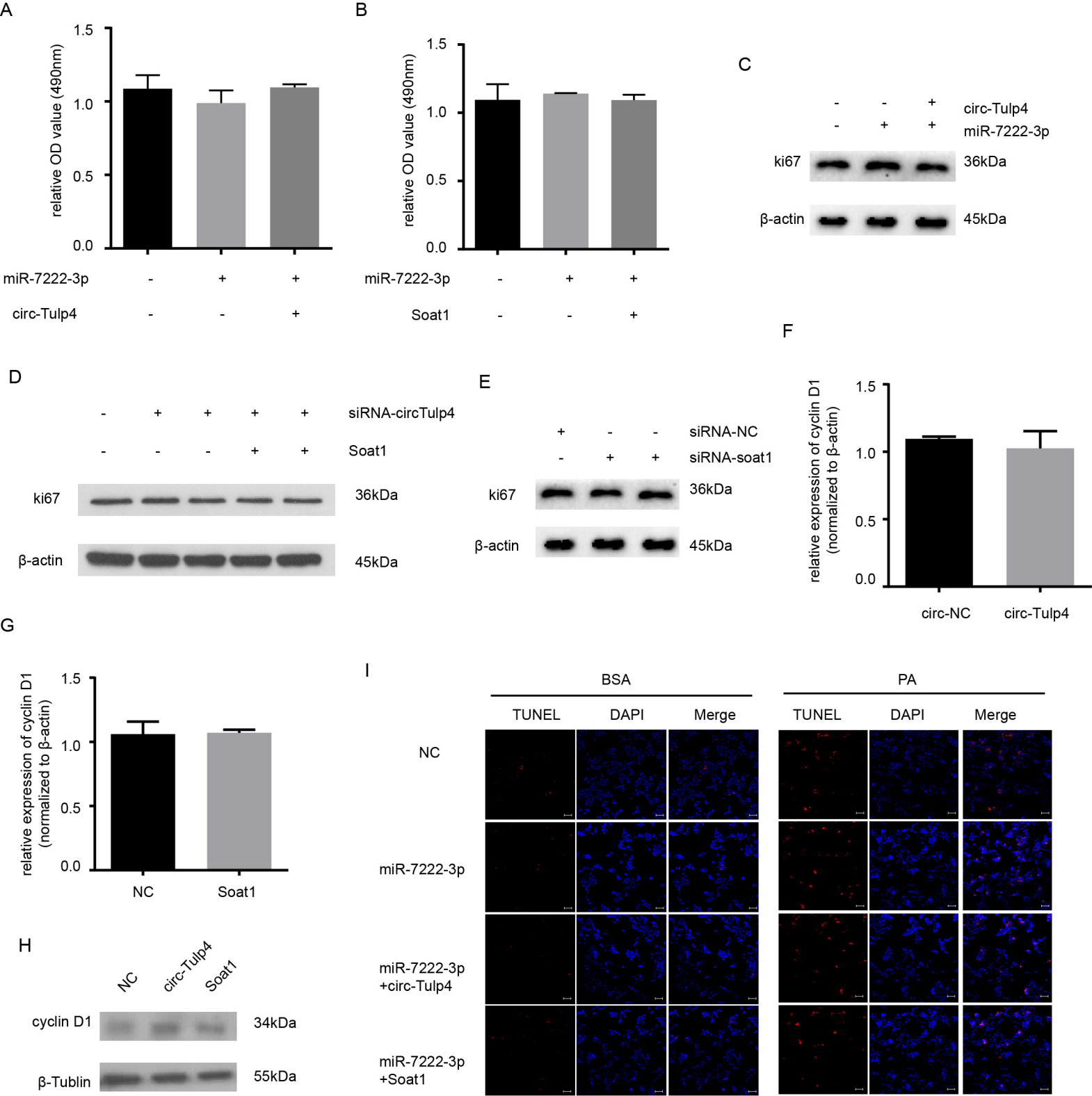

Supplement: Supplementary Fig. 4 Min6 cells were transfected with miR-7222-3p mimic, or co-treated with circ-Tulp4 vector (A) or Soat1 vector (B) for 48 h, followed by BSA treatment for 24 h. Cell proliferation ability was detected by MTS. Min6 cells were transfected with miR-7222-3p mimic, or co-treated with c [file supplementary_figure_4.pdf]
