# Supplementary material for: Circ-Tulp4 promotes β-cell adaptation to lipotoxicity by regulating soat1 expression
Source: J Mol Endocrinol. 2020 Sep 11;65(4):149–61. doi: 10.1530/JME-20-0079 (PMC7576671; doi:10.1530/JME-20-0079)
Supplement: Supplementary Table 1 primers used for qRT-PCR in this study. [file supplementary_table_1.pdf]

Supplementary method

RNA extraction, and qRT-PCR

Total RNA from the isolated islet cells or cultured Min6 cells was extracted using the TRIzol reagent (Invitrogen, America) according to the manufacturer's protocol. cDNA was synthesized using the RT reagent kit (TaKaRa, Japan). The circRNA and mRNA levels were determined by quantitative real-time reverse transcription-polymerase chain reaction (qRT-PCR) using TB Green (TaKaRa). The relative expression levels of the selected genes were normalized to β-actin mRNA levels. The quantification of microRNA expression was performed with a stern-loop real-time PCR miRNA kit (Ribobio, China). U6 was chosen as an internal control. All qRT-PCR assays were carried out on the LightCycler 480 Ⅱ real-time PCR detection system (Roche). *All primers used in this study were designed using Primer premier software (6.0) and listed in Supplementary Table 1.*

Verification of circularity by RNase R treatment

The total RNA was treated with RNase R (Geneseed, China) or RNase-free water (control), and digested RNA was used for confirmation.

RNA FISH

The circ-Tulp4 probes (Cy3-labeled) were designed and synthesized by Geneseed. The sequences of the probes were specific for covering the junction site of the circ-Tulp4 (sequences: gttgcacatctgatatctccatg). The *in situ* protocol was performed according to the instructions. 4',6-diamidino-2-phenylindole (DAPI) solution was used to detect nuclei. Images were acquired using the TCS SP2 AOBS confocal microscope system.

MTS assay

The Cell Titer 96 AQueous One Solution Cell Proliferation Assay System (MTS; Promega, America) was applied to detect cell survival according to the manufacturer's protocols. The absorbance at 490nm was measured using a microplate reader (Bio-Rad, America).

EdU assay

The cell proliferation rate was detected using EdU (5-ethynyl-2'-deoxyuridine) DNA Cell Proliferation Kit (RiboBio) according to the manufacturer's protocol. Hoechst 33342 was used to detect nuclei. Images were acquired using the inverted fluorescence microscope system (Olympus, Japan) and analyzed using Image-Pro Plus 6.0 software. For accurate assessment of the cell proliferation rate, the number of EdU-positive cells was also measured using a Beckman CytoFLEX cytometry system (Beckman).

Luciferase assay

The luciferase reporter was constructed by subcloning the circ-Tulp4 region containing predicted microRNA binding sites or soat1 3' untranslated region (3'-UTR) fragment directly downstream of the reporter gene Renilla luciferase into the psiCHECK2 luciferase vectors. Luciferase activity was determined using the Dual-Luciferase Reporter Assay Kit (Beyotime, China) following the manufacturer's protocol.

Apoptosis assay

The number of apoptotic cells was evaluated by using the Annexin V-FITC/PI apoptosis assay kit

(Lianke Biotech, China) according to the manufacturer's protocol. TUNEL staining was performed using the One step TUNEL apoptosis assay kit (Beyotime Biotech, China) according to the manufacturer's protocol. The rate of apoptosis was analyzed by ImageJ software.

Cell cycle analysis

The cell cycle was detected by propidium iodide (Invitrogen) staining according to the manufacturer's protocol. The percentage of cells was counted using a Beckman CytoFLEX cytometry system (Beckman).

Metabolic measurements

Bodyweight and food intake (g/day/body weight) were monitored periodically. Blood was taken from the tail vein to measure blood glucose using Accu-Chek Glucometer (Roche). Overnight fasting glucose of mice on a high-fat diet (HFD) above 10mmol/L were considered diabetic and used for experiments. Glucose tolerance tests were performed by an intraperitoneal injection of 2 g/kg glucose after overnight fasting.

Immunofluorescence

Freshly isolated islets were fixed and labeled with anti-insulin antibody (cat#3014 Cell Signaling Technology) to determine the cell type of isolated cells via the immunofluorescence method.

Measurement of cholesterol ester in Min6 cells

Min6 cells were overexpressed with circ-Tulp4 or soat1 for 48 h and then followed by PA treatment for 24 h. Cholesterol ester was evaluated using a cholesterol assay kit (BioVision, Milpitas, CA).

Supplementary figure legends

Supplementary Fig. 1

Determination of body weight, blood glucose, food intake and glucose tolerance in db/db mice and db/m mice (**A-D**), or in C57BL/6J mice on a normal control or high-fat diet (**F-H**). Bodyweight (**A**), food intake (g/day/body weight) (**B**), and random blood glucose measured using a glucometer (Roche) from 6 to 9 weeks of age (n≥10 in **A and C**, n=5 in **B**). **D** Intraperitoneal glucose tolerance testing at 10 weeks of age. The mice were fasted overnight, and the blood glucose levels were monitored in response to 2 g/kg glucose (n=5). Blood glucose levels at all time points were comparatively high in db/db mice versus db/m mice. Data represent mean ± standard error of the mean. ***, $P < 0.001$ versus db/m mice. **E** Representative images of freshly isolated mice islets and insulin staining images. Insulin immunofluorescence assay was performed to confirm that the cells used for RNA-seq were acinar-free islets. The results indicated that isolated cells were mostly stained positive. Plots of body weight (**F**) and fasting blood glucose (**G**) of C57BL/6J mice over time (n≥10). A plot of time-dependent glucose tolerance curves in 37-week old C57BL/6J mice on a normal control (NFD) or high-fat diet (HFD) (n≥10). Blood glucose levels at all time points were comparatively high in HFD mice versus NFD mice. ***, $P < 0.001$ versus C57BL/6J mice on a NFD.

Supplementary Fig. 2

Min6 cells were transfected with circ-Tulp4 siRNAs for 24 h (**A** and **C**) or 48 h (**B**), followed by PA (0.5mM) (**C**) or solvent (BSA) treatment for 24 h (**A** and **B**). Cell proliferation ability was detected by MTS under basal condition or lipotoxic condition. To examine cell proliferation under basal condition,

EdU assay (**D and E**) or western blot (**F**) was performed. Insulin biosynthesis (**G-H**) and apoptosis (**I-L**) were not affected by the silencing of circ-Tulp4. The protein expression level of cleaved caspase-3 was analyzed by Western blot under lipotoxic condition. (**I and J**). Min6 cells stained with Annexin V and propidium iodide (PI) were analyzed by flow cytometry for cell apoptosis assessment under basal (**K**) or lipotoxic (**L**) condition. *, $P < 0.05$ *versus* indicated groups.

Supplementary Fig. 3

To assess cell apoptosis, Min6 cells stained with Annexin V and propidium iodide (PI) were analyzed by flow cytometry (**A-B**). Expression of insulin1 mRNA (ins1) or insulin2 mRNA (ins2) was analyzed by qRT-PCR under lipotoxic condition after upregulating circ-Tulp4 (**C**) or soat1 (**D**) expression. Cell survival was examined by MTS in the siRNA-soat1 transfected cells (**E**) or Soat1 vector-infected cells (**F**) under basal condition. MiR-298-5p, miR-3113-3p, and miR-7222-3p demonstrated a potentially relevant role in regulating the expression of soat1, and verification of these microRNAs expressions in Min6 cells was shown (**G**). MiR-3113-5p served as a control. Expression level of soat1 in Min6 cells treated with either miR-298-5p mimic or co-treated with miR-298-5p mimic and circ-Tulp4 vector (**H**). Expression level of soat1 in Min6 cells treated with either miR-3113-3p mimic or co-treated with miR-3113-3p mimic and circ-Tulp4 vector (**I**). NS, Non-significance of difference. *, $P < 0.05$; **, $P < 0.01$ *versus* the indicated groups.

Supplementary Fig. 4

Min6 cells were transfected with miR-7222-3p mimic, or co-treated with circ-Tulp4 vector (**A**) or Soat1 vector (**B**) for 48 h, followed by BSA treatment for 24 h. Cell proliferation ability was detected

by MTS. Min6 cells were transfected with miR-7222-3p mimic, or co-treated with circ-Tulp4 vector for 48 h(**C**); or transfected with siRNA-1 or siRNA-2 for circ-Tulp4, or co-treated with Soat1 vector for 48 h (**D**); or transfected with siRNA-1 or siRNA-2 for soat1 for 48 h (**E**), followed by BSA treatment for 24 h. Western blot assays were used to analyze the protein expression level of ki67. The expression level of cyclin D1 mRNA (**F and G**) or protein (**H**) in Min6 cells infected with circ-Tulp4 or Soat1 vector was analyzed. For apoptosis assessment, TUNEL staining was performed and TUNEL positive Min6 cells with indicated treatment were counted (**I**). Scale bar = 50 μm. Non-significant differences were observed in the above groups.

Supplementary Table 1 primers used for qRT-PCR in this study.

Gene	Primer sequences

circ-Tulp4	F: AATAAACTTCAACCTGCGAGGC
	R: CCGGTTAATTCAGGAGCCATC
tulp4 mRNA	F: AATAAACTTCAACCTGCGAGGC
	R: CCGGTTAATTCAGGAGCCATC
soat1 mRNA	F: TCGCACTCCTCATCCTAT
	R: TCAAGTACCAGCCTTCCT
cyclin D1 mRNA	F: GGAGCAGAAGTGCGAAGA
	R: CAGTCAAGGGAATGGTCTC
β-actin mRNA	F: GACGGCCAGGTCATCACTATTG

	R: CCACAGGATTCCATACCCAAGA

AccID	Chrom	start	end	strand	Gene Name	$\log^2$(fold change)

Supplementary Table 2 detailed information of the differentially expressed circRNAs with a fold change over 1.5.

chr6_28545598_28526053_+19545-Snd1	chr6	28526052	28545598	+	Snd1	4.415336611
chr4_138241792_138221526_+20266-Hp1bp3	chr4	138221525	138241792	+	Hp1bp3	3.870871984
chr18_75082507_75080203_+2304-Dym	chr18	75080202	75082507	+	Dym	3.304441839
chr4_108719812_108717789_-2023-Zfyve9	chr4	108717788	108719812	-	Zfyve9	3.047468137
chr15_73031643_72999972_-31671-Trappc9	chr15	72999971	73031643	-	Trappc9	2.846222385
chr9_84012619_83988776_+23843-Bckdhb	chr9	83988775	84012619	+	Bckdhb	1.444980597
chr15_95920378_95864225_+56153-Ano6	chr15	95864224	95920378	+	Ano6	1.384757284
chr1_38085652_38053616_-32036-Rev1	chr1	38053615	38085652	-	Rev1	1.323683763
chr17_6139156_6137211_+1945-Tulp4	chr17	6137210	6139156	+	Tulp4	-0.815422012
chr18_6115850_6111685_-4165-Arhgap12	chr18	6111684	6115850	-	Arhgap12	-2.404150957
chr1_87380008_87364104_+15904-Gigyf2	chr1	87364103	87380008	+	Gigyf2	-2.776675445
chr16_94403368_94383912_+19456-Ttc3	chr16	94383911	94403368	+	Ttc3	-3.017806158

chr5_135124017_135121506_+2511-Mlxipl	chr5	135121505	135124017	+	Mlxipl	-3.0186879
chr1_155962560_155953154_-9406-Cep350	chr1	155953153	155962560	-	Cep350	-3.810876653

+： sense strand; -: antisense strand; mouse gene information (mm10) was used as reference for alignment.

Sequence of mouse circ-Tulp4

5 ‘-AGTTGTAAGAGTCCATCCAGGACCTTCCAGTCATGAATAATCTGATGGCTCCTGAATTAA

CCGGGAAAACAAACATATCAAGTGCCATTTGAAGACTCTGTCTATCTATGTAAAACCTTT

TCTGCACATAGAAGCTTTTCCATAAGAAGACATTCTGAATTTTGCAACTGATGAAGATTA

AGCATCAGCTGGAGCACCTTTCCACTGTTGGGGTTGGGAGTCTGTGGAGAACAGTCTGTC

ACTAATGTCAGATTTTCCTTACAGTGTTCATAAACAAAAGCCAGTTTGCAAAAGAAAAAA

TTGCACAGATTAACCCTAAAGAATAGCTCCAGTGTAAAGCAGGGGCAGACCTTAAAACTC

TGAACCGGAGCTCAGTGACTTTTCCTGTGGTTGTAGCAGGAGTGAGGGGACTGATCTGAA

AGGAACAGATTCCTTTGTGTCTTCAGCTATCGGAAGTTTTTTATTTATTGTTTATCTTTT

TTCTTTTCTGCATATATATGCCATTTTAAAATACTAATTGGAACTGTCAGTTACAAAATA

AATATCAAGAAAAGCCTTTTTTGGTCCAAAGGTGTGAACAGGCTTGCAGGTGAACAGGAA

GACATCTTTGGTAAAGCTTGGACCGGTCTTGGGAGATGGTGCATCTTGGAGGGCTCTGCT

ACACGCCTAGGTGGCTGGTTTAAAGGCTTGCATCGCAGACGGAGACTAATTAATAGACAC

AGTTCTAAGATTGCTTTTTCATTAACATAGAAACTAGAGAAAGGAGAAACAGAAGCCTGC

AGCCTAGTCTGTGTAAGCAAGAAGTAGGACCAAACTGAGGAAACTACTTGGTCCATTTAG

ATAGCAGTTTATTCATACTCAGTGACCCGCAGGCCTCCACCTCTGCTTGAGGGAAGGGCT

TTGCTCCAGTCTCTGTGGCACTGAGGGTGGTTCCAGCCCATGGAGGAGTCATTCTAGGAA

GCCCTGTGTTCCTAGGGACACAGGGCCAGGCTTTGAGACAGGAAGCTTCTGGCTGTGAGC

AGTGGGGGAAAGAGTGATTTTCTTGTTAAAGCTTTGACCATTGTCTGCATGAGCTCTGGT

GTGACTTTGCACGTTAGTGTGCCTTTCCCCTTATGCAACCTTTTCCAGCTTACAGCAGAA

ACTTGCCGAGTTCAGAAAACGTGCCAGAGGGTGGCTTCAGAGGGAAGATGATCTTGTGTG

ATCAGTCTCTGCACTTGAACTATTGAATAGAGAAATCCAGCTAGAGGAATTCTTACCGCC

TTAAGTTACTTGAAATCTATGTGTTTGTAACCCTTTGTCTCTGGAATTACATTACAAAAA

AAACTGGAATCTCAGGCTGAGAATAACGAGGCTGAGTAAAAGCGAAGAGAACTGCCTCTT

CATCATCACTTACTAACAGCTCTTTCTCCAAAGGATTGGTGTGGTTTCCCGCTAAGAACT

TGAAAATGAGAACGGACCCTGTGTATTTTTAGGCATTACCTTTCTTCGCCGACTGACGTC

TTTTATAGAGGAGTTTTTTCACTATGCATTTGGTGGAGCTTTATAAGCTATTGACCTAAT

TGGACTCTAGATCAGTTGTAACTAAAGGAGAAAAAAACAAACCAACGGAACCCAACCACA

AAAATAAGCCAATAAAAGAACTTGGTTTGAAATTCCTCAGTACTTTTAAAGTGAAATACT

TCATTGAAAAAAGTATGTATGCAGCAGTGGAACATGGGCCTGTGCTTTGCAGCGATTCCA

ACATCCTCTGCCTGTCCTGGAAGGGGCGTGTTCCCAAGAGTGAGAAGGAGAAACCTGTGT

GCAGAAGGCGCTACTATGAAGAGGGATGGTTGGCCACAGGCAATGGGCGAGGTGTGGTGG

GAGTGACTTTCACCTCGAGTCACTGTCGCAGAGATAGGAGTACACCACAGAGAATAAACT

TCAACCTGCGAGGCCACAACAGTGAG-3’

Sequence of relevant human circ-Tulp4

5’-ATTTGTAAGACTCCAGGGCCTCCCAGCCGTGAATAATCTGATGGTTCCTGAAATGACTGGGGAAGCAGACGCTTCGTATG

GCAGTTGAAGAGTGTGTGTCTATGTGCATTTAAAACCTTCTTTCTGTACTTACACATTCACACGGGAAGACAGGCTCATT

CTTGTGCACACTTGAGAGTTTTACAACTGATGAAAATTAATTTAAGAATCAGATGGAGCAACTTGACACCAGTGGGCTCA

GGAGCCCGGGGAGAAAAATACATCACTAATGGCCAGTTTTCCATATGGTCTGCACGGGTAAAGAAAGTCTGCAAAAAGAA

GAAAAAAAAATTTGCGCAGATTAAACCACAAAAATATTCTCCAGTTTAAAGAAGGAACTAAGTGAGAAGGTGACTGAGAA

AGAAGTGTGATTTCAAACATTGCAGCGGCTCACACAGTGTTTGTTGCACTTTATTTTTCAGTGGGTTTGGTGATTTGGAC

GGATTAAAATTCTAGACTGAAAAGTAACTCCTACTGTGGTTATGGCTAGAGGAAAGCAAGTTCAAGTATGATGGGACAAG

TTTGAATAATGAACTGATTCCTTTGCCTATCTTAATTAACTGTATTTGAGAAATTTTAATTTATTATTCCCCCCCTTTTT

TCCTGCATCTATAGGATAATATTGTAAAATAGCAATTGAAACCAATAATTATTAAATAAATATCAAGGAAAATCCAAGCA

AAGCTTTCTTTTTGTTGGACTAGTGGTGTGGTGTTTGGAGACAGTCTCTGAATGTGAACAGGAAAGCACCCATCAGCAAA

ACACTATCACTCTCTAGGGAGACAGCTGGGGGAATCTGACTCTGGCTTCTGCTTTTGTTTTAAGGGATTAACTTCCCTGT

CAAGTCCAAGAAGACTTGCGTATGAGAAGATTACCTGATGGACTTAATTCTAAGATTAGCTTTTTTCATCAAGATGGAAA

AAGATCTTTAGGAGCAGAAAAGGGGAGTGCTAACTGGGGGAGCGAGAAGGGAGACGAGCAAAAGAAACAAAATCTTGCCA

CGTGGCTCTGTTTTGTCAGCAAGAGGATTTAAGACTCACCCAGGGCAAACACTGGGACCACTGTAAGAGCGCTGGAACAT

TCTGCCTCTTGAGTGAAGGGGCCTTCTTTCTAGCCTCTATGGCACTGAGGGGTGCGCCGGCTGGTGGAGGAGCAGTCCGA

TGGAGCCCTGCGTTCCCCGGGGACACAGGGCCAAGCTTTGAGGTGGAAAGTTTCTGGTTCTGAAACAACAAGGAGAGAGT

CTGTTTTTCTTCCTAAAATTTGGACTCTTGTCTGCACAAACTCTGGTCTGTTTTGCACGGTTTGTGTGCCTTTTTTTCCC

TTTATGCAATCTTTTTCAGCTTTAGCAGCAGAAATTTGTCTAGTTCAGGAAACATGCTAGAGGGTGGCTTCAGAAGGAAG

ATGATCCTGTGTATTCTGTCTCTGCATCCGAACTTTTGAAGAGAAAAATTCGAGCTAGAGGGATTCTTAAAGCCTTAAGT

TACTTGAAATCTATGTATTTGCAACCCTTTGTCTCTGGAATCATATTACACTAAACTGGAATCTCAGGCTGAATGAGAAT

AACCAAGTGGAGTAAAAAGAAGAAAACCGTTTCTTGATCACCACTTAATTAACGATGCTCTTTCTCCAAAGGATCAGCAC

GTTCTTCCTCTGAGAACTTGAAAATACAAATGGACCCCATGTTTTTTTAAGCATTACCTTTTCTTAGAAGACTGCCATCA

TCTTTTATAGAGGAATTTTTTCACTATGCATTCGGTGGATCTTTATAAAATACTGACCTTCTAATTAGATTCAGGTCAGT

CTTAATTAAAGGGGGAAAAAAGCAACGCAAGCCAACCACAAAAACACATATACCAATGAAAGAAATTGGTTTAAATTTCA

CAGCATTAACATTACTTTTTAAGTAAAACAGTTCATTGAAGAAAGTATGTATGCAGCAGTGGAACATGGGCCTGTGCTTT

GCAGCGATTCCAACATCCTGTGCCTGTCCTGGAAGGGGCGTGTCCCCAAGAGTGAGAAGGAGAAGCCTGTGTGCAGGAGA

CGCTACTATGAGGAAGGCTGGCTGGCCACGGGCAACGGGCGAGGAGTGGTTGGGGTGACTTTCACCTCTAGTCACTGTCG

CAGGGACAGGAGTACTCCACAGAGGATAAATTTCAACCTCCGGGGCCACAATAGCGAG-3’

Cross-species comparison is shown in another PDF supplementary material using the MUSCLE tool (https://www.ebi.ac.uk/Tools/msa/muscle/). *, conserved nucleotides.
