# Supplementary material for: Circ-Tulp4 promotes β-cell adaptation to lipotoxicity by regulating soat1 expression
Source: J Mol Endocrinol. 2020 Sep 11;65(4):149–61. doi: 10.1530/JME-20-0079 (PMC7576671; doi:10.1530/JME-20-0079)
Supplement: Supplementary Materials and methods [file supplementary_material.pdf]

Results for job muscle-I20200301-015410-0379-9811389-p2m

```
CLUSTAL multiple sequence alignment by MUSCLE (3.8)


human_circ-Tulp4      ATTTGTAAGA----CTCCAGGGCCTCCCAGCCGTGAATAATCTGATGGTTCCTGAAATGA
mouse_circ-Tulp4      AGTTGTAAGAGTCCATCCAGGACCTTCCAGTCATGAATAATCTGATGGCTCCTGAATTAA
                      * ********     ****** *** **** * *************** ******* * *

human_circ-Tulp4      CTGGGGAAGCAGACGCTTCGTATGGCAGTTGAAGAGTGTGTGTCTATGTGCATTTAAAAC
mouse_circ-Tulp4      CCGGGAAAACAAACATATCAAGTGCCATTTGAAGACTCTGTCTATCTATG----TAAAAC
                      * *** ** ** **   **   ** ** ******* * *** * * * **    ******

human_circ-Tulp4      CTTCTTTCTGTACTTACA--CATTCACACGGGAAGACAGGCTCATTCTTGTGCACACTTG
mouse_circ-Tulp4      CT--TTTCTGCACATAGAAGCTTTTCCATAAGAAGA------CATTCT------------
                      **  ****** ** ** *  * **  **   *****      ******

human_circ-Tulp4      AGAGTTTTACAACTGATGAAAATTAATTTAAGAATCAGATGGAGCAACTTGACACCAGTG
mouse_circ-Tulp4      -GAATTTTGCAACTGATGAAGA-----TTAAGCATCAGCTGGAGCACCTTTCCACTGTTG
                       ** **** *********** *     ***** ***** ******* ***  ***   **

human_circ-Tulp4      GGCTCAGGAGCCCGGGGAGAAAAATACATCACTAATGGCCAGTTTTCCATATGGTCTGCA
mouse_circ-Tulp4      GGGTTGGGAGTCTGTGGAGAACAGTCTGTCACTAATGTCAGATTTTCCTTACAGTGTTCA
                      ** *  **** * * ****** * *   ********* *   ****** **  ** * **

human_circ-Tulp4      CGGGT-AAAGAAAGTCTGCAAAAAGAAGAAAAAAAAATTTGCGCAGATTAAACCACAAAA
mouse_circ-Tulp4      TAAACAAAAGCCAGTTTGCA--------AAAGAAAAAATTGCACAGATTAACCCTAAAGA
                            ****  *** ****        *** ***** **** ******** **  ** *

human_circ-Tulp4      ATATTCTCCAGTTTAAAGAAGGAACTAAGTGAGAAGGTGACTGAGAAAGAAGTGTGATTT
mouse_circ-Tulp4      ATA-GCTCCAGTGTAAAGCAGGGGCAGACC------------------------------
                      ***  ******* ***** ***  *  *

human_circ-Tulp4      CAAACATTGCAGCGGCTCACACAGTGTTTGTTGCACTTTATTTTTCAGTGGGTTTGGTGA
mouse_circ-Tulp4      ------------------------------------------------------------


human_circ-Tulp4      TTTGGACGGATTAAAATTCTAGACTGAAA---AGTAACTCCTACTGTGGTTATGGCTAGA
mouse_circ-Tulp4      ----------TTAAAACTCTGAACCGGAGCTCAGTGACTTTTCCTGTGGTTGTAGCAGGA
                                ****** ***  ** * *    *** ***  * ******** * **  **

human_circ-Tulp4      GGAAAGCAAGTTCAAGTATGATGGGACAAGTTTGAATAATGAACTGATTCCTTTGCCTAT
```

```
mouse_circ-Tulp4      G-----------------TGAGGGGACTGATCTGA--AAGGAACAGATTCCTTTG--TGT
                      *                 *** *****   * ***  ** **** **********  * *

human_circ-Tulp4      CTTAATTAACTGTATTTGAGAAATTTTAATTTATTATTCCCCCCCTTT-TTTCCTGCATC
mouse_circ-Tulp4      CTT------CAGCTATCGGAAGTTTTTTATTTATTGTTTATCTTTTTTCTTTTCTGCATA
                      ***      * *   * *  *  **** ******* **   *   *** *** ******

human_circ-Tulp4      TATAGGATAATATTGTAAAATAGCAATTGAAACCAATAATTATTAAATAAATATCAAGGA
mouse_circ-Tulp4      TAT---ATGCCATTTTAAAATACTAATTGGAACTGTCAGTTACAAAATAAATATCAAGAA
                      ***   **   *** *******  ***** ***    * ***  ************** *

human_circ-Tulp4      AAATCCAAGCAAAGCTTTCTTTTTGTTGGACTAGTGGTGTGGTGTTTGGAGACAGTCTCT
mouse_circ-Tulp4      AAGCC----------------TTTTTTGGTCCAAAGGTGTG---------AACAGGCT-T
                      **  *                *** **** * *  ******          **** ** *

human_circ-Tulp4      GAATGTGAACAGGAAAGCACCCATCAGCAAAACACTATCACTCTCTAGGGAGACAG----
mouse_circ-Tulp4      GCAGGTGAACAGGAAGACATCT-TTGGTAAAGC-TTGGACCGGTCTTGGGAGATGGTGCA
                      * * ***********  ** *  *  * *** *  *    *  *** ******  *

human_circ-Tulp4      --CTGGGGGAATCTGACTCTGGCTTCTGCTTTTGTTTTAAGGGATTAACTTCCCTGTCAA
mouse_circ-Tulp4      TCTTGGAGGGCTCTGCTACACGCCTAGGTGGCTGGTTTAAAGGCTTGCAT----------
                         *** **  ****   *  ** *  *    ** ***** ** **   *

human_circ-Tulp4      GTCCAAGAAGACTTGCGTATGAGAAGATTACCTGATGGACTTAATTCTAAGATTAGCTTT
mouse_circ-Tulp4      ---------------CGCAGACGGAGACTAATTAATAGACACAGTTCTAAGATTG--CTT
                                     ** *   * *** **  * ** ***  * **********    **

human_circ-Tulp4      TTTCATCAAGATGGAAAAAGATCTTTAGGAGCAGAAAAGGGGAGTGCTAACTGGGGGAGC
mouse_circ-Tulp4      TTTCATTAACATAGAAA-----------------------------CTA-----------
                      ****** ** ** ****                             ***

human_circ-Tulp4      GAGAAGGGAGACGAGCAAAAGAAACAAAATCTTGCCACGTGGCTCTGTTTTGTCAGCAAG
mouse_circ-Tulp4      GAGAAAGG-----------AGAAACAGAAGCCTGCAGCCTAG-TCTG---TGTAAGCAAG
                      ***** **           ******* ** * ***  * * * ****   *** ******

human_circ-Tulp4      AGGATTTAAGACTCACCCAGGGCAAACACTGGGACCACTGTAAGAGCG------------
mouse_circ-Tulp4      AAG---TAGGACCAAACTGAGGAAACTACTTGGTCCATTTAGATAGCAGTTTATTCATAC
                      * *   ** ***  * *   ** **  *** ** *** *   * ***

human_circ-Tulp4      --------CTGGAACATTCTGCCT---CTTGAGTGAAGGGGCCTTCTTTCTAGCCTCTAT
mouse_circ-Tulp4      TCAGTGACCCGCAGGCCTCCACCTCTGCTTGAGGGAAGGG--CTTTGCTCCAGTCTCTGT
                              * * *    **  ***   ****** ******  ***   ** ** **** *

human_circ-Tulp4      GGCACTGAGGGGTGCGCCGGCTGGTGGAGGAGCAGTCCGATGGAGCCCTGCGTTCCCCGG
mouse_circ-Tulp4      GGCACTGAGGGTGGTTCCAGCCCATGGAGGAGTCATTCTAGGAAGCCCTGTGTT-CCTAG
                      ***********  *  ** **   ********   * * * * ******* *** **  *

human_circ-Tulp4      GGACACAGGGCCAAGCTTTGAGGTGGAAAGTTTCTGGTTCTGAAACAACAAGGAGAGAGT
mouse_circ-Tulp4      GGACACAGGGCCAGGCTTTGAGACAGGAAGCTTCTGGCTGTGAGCAGTGGGGGAAAGAG-
                      ************* ********   * *** ****** * ***        *** ****

human_circ-Tulp4      CTGTTTTTCTTCCTAAAATTTGGACTCTTGTCTGCACAAACTCTGGTCTG-TTTTGCACG
mouse_circ-Tulp4      -TGATTTTCTTGTTAAAGCTTTGACCATTGTCTGCATGAGCTCTGGTGTGACTTTGCAC-
                       ** *******  ****  ** ***  *********  * ******* **  *******

human_circ-Tulp4      GTTTGTGTGCCTTTTTTTCCCTTTATGCAATCTTTTTCAGCTTTAGCAGCAGAAATTTGT
mouse_circ-Tulp4      GTTAGTGTGCC----TTTCCCCTTATGCAACCTTTTCCAGCTT--ACAGCAGAAACTTGC
                      *** *******    ****** ******** ***** ******   ********* ***

human_circ-Tulp4      CTAGTTCAGGAAACATGCTAGAGGGTGGCTTCAGAAGGAAGATGATCCTGTGTATTCTGT
mouse_circ-Tulp4      CGAGTTCAGAAAACGTGCCAGAGGGTGGCTTCAGAGGGAAGATGATCTTGTGTGATCAGT
                      * ******* **** *** **************** *********** *****  ** **

human_circ-Tulp4      CTCTGCATCCGAACTTTTGAAGAGAAAAATTCGAGCTAGAGGGATTCTTAAAGCCTTAAG
mouse_circ-Tulp4      CTCTGCACTTGAACTATTGAATAGAGAAATCC-AGCTAGAGGAATTCTTACCGCCTTAAG
                      *******   ***** ***** *** **** * ********* *******  ********

human_circ-Tulp4      TTACTTGAAATCTATGTATTTGCAACCCTTTGTCTCTGGAATCATATTAC--ACTAAACT
mouse_circ-Tulp4      TTACTTGAAATCTATGTGTTTGTAACCCTTTGTCTCTGGAATTACATTACAAAAAAAACT
```

```
                      ***************** **** ******************* * *****  *  *****

human_circ-Tulp4      GGAATCTCAGGCTGAATGAGAATAACCAAGTGGAGTAAAAA-GAAGAAAACCGTTTCTTG
mouse_circ-Tulp4      GGAATCTCAGGC----TGAGAATAACGAGGCTGAGTAAAAGCGAAGAGAACTGCCTCTTC
                      ************    ********** * *  ********  ***** *** *  ****

human_circ-Tulp4      ATCACCACTTAATTAACGATGCTCTTTCTCCAAAGGATCAGCACGTTCTTCCTCTGAGAA
mouse_circ-Tulp4      ATCATCACTT-ACTAACA--GCTCTTTCTCCAAAGGATTGGTGTGGTTTCCCGCTAAGAA
                      **** ***** * ****   ******************  *   * * * ** ** ****

human_circ-Tulp4      CTTGAAAATACAAATGGACCCCATGTTTTTTTAAGCATTACCTTTTCTTAGAAGACTGCC
mouse_circ-Tulp4      CTTGAAAATGAGAACGGACCCTGTGTATTTTTAGGCATTACC-TTTCTTCGCCGACTGA-
                      *********   ** ******  *** ****** ******** ****** *  *****

human_circ-Tulp4      ATCATCTTTTATAGAGGAATTTTTTCACTATGCATTCGGTGGATCTTTATAAAATACTGA
mouse_circ-Tulp4      --CGTCTTTTATAGAGGAGTTTTTTCACTATGCATTTGGTGGAGCTTTATAAGCTATTGA
                        * ************** ***************** ****** ********  ** ***

human_circ-Tulp4      CCTTCTAATTAGATTC-AGGTCAGTCTTAATTAAAGGGGGAAA----AAAGCAACGCAAG
mouse_circ-Tulp4      C---CTAATTGGACTCTAGATCAGTTGTAACTAAAGGAGAAAAAAACAAACCAACGGAAC
                      *   ****** ** ** ** *****  *** ****** * ***    *** ***** **

human_circ-Tulp4      CCAACCACAAAAACACATATACCAATGAAAGAAATTGGTTTAAATTTCACAGCATTAACA
mouse_circ-Tulp4      CCAACCACAAAA----ATAAGCCAATAAAAGAACTTGGTTTGAAATTCCT--------CA
                      ************    ***  ***** ****** ******* ** ***          **

human_circ-Tulp4      TTACTTTTTAAGTAAAACAGTTCATTGAAGAAAGTATGTATGCAGCAGTGGAACATGGGC
mouse_circ-Tulp4      GTACTTTTAAAGTGAAATACTTCATTGAAAAAAGTATGTATGCAGCAGTGGAACATGGGC
                       ******* **** *** * ********* ******************************

human_circ-Tulp4      CTGTGCTTTGCAGCGATTCCAACATCCTGTGCCTGTCCTGGAAGGGGCGTGTCCCCAAGA
mouse_circ-Tulp4      CTGTGCTTTGCAGCGATTCCAACATCCTCTGCCTGTCCTGGAAGGGGCGTGTTCCCAAGA
                      **************************** *********************** *******

human_circ-Tulp4      GTGAGAAGGAGAAGCCTGTGTGCAGGAGACGCTACTATGAGGAAGGCTGGCTGGCCACGG
mouse_circ-Tulp4      GTGAGAAGGAGAAACCTGTGTGCAGAAGGCGCTACTATGAAGAGGGATGGTTGGCCACAG
                      ************* *********** ** *********** ** ** *** ******* *

human_circ-Tulp4      GCAACGGGCGAGGAGTGGTTGGGGTGACTTTCACCTCTAGTCACTGTCGCAGGGACAGGA
mouse_circ-Tulp4      GCAATGGGCGAGGTGTGGTGGGAGTGACTTTCACCTCGAGTCACTGTCGCAGAGATAGGA
                      **** ******** ***** ** ************** ************** ** ****

human_circ-Tulp4      GTACTCCACAGAGGATAAATTTCAACCTCCGGGGCCACAATAGCGAG
mouse_circ-Tulp4      GTACACCACAGAGAATAAACTTCAACCTGCGAGGCCACAACAGTGAG
                      **** ******** ***** ******** ** ******** ** ***
```
